# A tale of two lineages: comparative biogeography across the Southern Cone of two South American *Carex* (Cyperaceae) groups

**DOI:** 10.1093/aob/mcaf262

**Published:** 2025-10-22

**Authors:** Ana Morales-Alonso, Paulo Muñoz-Schüler, Tamara Villaverde, Pedro Jiménez-Mejías

**Affiliations:** Área de Biodiversidad y Conservación, Departamento de Biología, Geología, Física y Química Inorgánica, Universidad Rey Juan Carlos, C/ Tulipán s/n, Móstoles E-28933, Madrid, Spain; Instituto de Investigación en Cambio Global, Universidad Rey Juan Carlos, C/Tulipán s/n, Móstoles E-28933, Madrid, Spain; Área de Botánica, Departamento de Biología Molecular e Ingeniería Bioquímica, Universidad Pablo de Olavide, Ctra. de Utrera, Km 1, Seville E-41013, Spain; Herbario CONC, Departamento de Botánica, Facultad de Ciencias Naturales y Oceanográficas, Universidad de Concepción, Casilla 160-C, Concepción, Chile; Área de Biodiversidad y Conservación, Departamento de Biología, Geología, Física y Química Inorgánica, Universidad Rey Juan Carlos, C/ Tulipán s/n, Móstoles E-28933, Madrid, Spain; Instituto de Investigación en Cambio Global, Universidad Rey Juan Carlos, C/Tulipán s/n, Móstoles E-28933, Madrid, Spain; Área de Botánica, Departamento de Biología Molecular e Ingeniería Bioquímica, Universidad Pablo de Olavide, Ctra. de Utrera, Km 1, Seville E-41013, Spain

**Keywords:** Andes Cordillera, biogeography, dry diagonal, eSADD, Hyb-Seq, wSADD

## Abstract

**Background and Aims:**

The *Carex phalaroides* group and *Carex* sect. *Bracteosellae* are two species complexes that exhibit similar distribution patterns, despite being two evolutionarily independent lineages. Their centre of diversity is displayed on the eastern coast of the Southern Cone, with disjunct lineages that have successfully colonized and diversified in central Chile and throughout the central and northern Andes. Given the scarce research about the biogeography of herbaceous lineages in the Southern Cone, our aim is to elucidate their evolutionary trajectories and compare their biogeographical histories from a macroecological perspective, considering the major geoclimatic events in these regions.

**Methods:**

We conducted Hyb-Seq phylogenomic analyses for both groups. We followed a bioclimatic approach to trace their historical biogeography across South America; thus, we estimated the divergence times, reconstructed their ancestral areas and characterized their ecological niches, inferring their climatic preferences over time.

**Key Results:**

The *C. phalaroides* group displayed a more evident vicariant pattern than sect. *Bracteosellae* on both sides of the Arid Diagonals during the earliest cladogenetic events. Both groups displayed synchronic diversification processes, specifically regarding movements between the Pampa–Atlantic region, colonization of the northern Andes and differentiation within the Yungas. Bioclimatic analyses retrieved a clear separation between eastern and western lineages within the *C. phalaroides* group, with *C. via-montana* exhibiting a distinct shift in temperature-related variables. In contrast, this geographical structuring of bioclimatic preferences was not observed in sect. *Bracteosellae*.

**Conclusions:**

We identified several geoclimatic events as key drivers of diversification. The Mid-Miocene Climatic Optimum and subsequent marine transgressions probably facilitated the expansion of the *C. phalaroides* group ancestor beyond Patagonia. Both South American Dry Diagonals acted as major barriers, fragmenting a once broader distribution and promoting speciation through vicariance. The Andes Cordillera functioned as a south-to-north biological corridor for both Andean lineages. Following vicariance and isolation, both groups evolved broad ecological niches, reflecting adaptive specialization to diverse environments.

## INTRODUCTION

Biodiversity patterns are shaped by the combined action of processes that operate at different scales and are driven by biotic (e.g. species interactions, intrinsic ecological niches, dispersal abilities) and abiotic factors (e.g. bioclimatic factors, geomorphological events) (Antonelli and Sanmartín, 2011). Understanding and studying how these patterns have evolved through space and time, shaping the current structure of biodiversity, is the main purpose of biogeography (Lomolino *et al.*, 2016). In this context, South America has traditionally aroused great interest among naturalists and scientists, as it is the continent with the greatest diversity of amphibians, birds and vascular plants (Myers *et al.*, 2000; Ulloa-Ulloa *et al.*, 2017). Most of this diversity is concentrated within the Neotropical region (*sensu*  Morrone *et al.*, 2022), which extends northwards into Mesoamerica and encompasses significant biodiversity hotspots such as the tropical Andes, the Atlantic Forest or the Tumbes–Chocó–Magdalena ecoregion (Antonelli *et al.*, 2018; Meseguer *et al.*, 2022; Pérez-Escobar *et al.*, 2022 ). Collectively, these regions harbour a disproportionate share of the continent’s overall diversity, in stark contrast to the comparatively less biodiverse neighbouring areas.

### The Southern Cone

Encompassing roughly the southern half of the continent, the Southern Cone is defined by Argentina, Chile, Paraguay, Uruguay and the Brazilian southernmost states of Paraná, Santa Catarina and Rio Grande do Sul (according to Zuloaga *et al.*, 2019). This region exhibits the greatest climatic and physiognomic variation on the continent, covering 9 out of the 14 biomes recognized by Olson *et al.* (2001) and ∼25 ecoregions, ranging from hyper-arid deserts to tropical rainforests and temperate and cold grasslands. It may be divided into two main areas: a south-western portion that encompasses the Andean region and the Patagonian region (Morrone, 2015), and a north-eastern half that belongs to the Neotropical region (Morrone *et al.*, 2022). This landscape heterogeneity is largely determined by a latitudinal north-to-south temperature gradient, related to the meridional projection of South America, and by striking east–west precipitation asymmetries driven by orographic and tropospheric features (Garreaud *et al.*, 2009).

The Andes Cordillera is the mountainous system that runs through the western side of South America from Venezuela to southern Chile and Argentina (Nagy and Grabherr, 2009; Körner *et al.*, 2011). It is usually divided into three geographical units (Luebert and Weigend, 2014): (1) the northern Andes, from Venezuela to northern Peru; (2) the central Andes, from north of Peru to the dry Puna (25–29° S); and (3) the southern Andes, entirely within the Southern Cone, extending from 29° S to the southernmost part where elevation declines. As the longest continental mountain range in the world, the Andes plays a crucial role in shaping the climate of South America in general and the Southern Cone in particular. Thus, the Andes act as a barrier to continental-scale air circulation, producing marked asymmetries in rainfall between the western and eastern slopes at different latitudes (Antonelli *et al.*, 2009; Garreaud *et al.*, 2009 ; Luebert and Weigend, 2014).

The Pampa region occupies the plains of central–eastern Argentina, Uruguay and southern Brazil (Baez-Lizarazo *et al.*, 2024). The temperate–subhumid region is dominated by temperate and subtropical grassland and shrubland, forming a heterogeneous landscape with a higher rate of species per unit than the entire Southern Cone (Andrade *et al.*, 2018; Oyarzabal *et al.*, 2020; Baez-Lizarazo *et al.*, 2024). Another shrub–herbaceous-dominated ecosystem relevant to this study is the Patagonian Steppe. Unlike the Pampa, the Patagonian Steppe is a semi-arid, cold region with a continental climatic that extends from Atlantic southern Argentina to south-western Chile (Gajardo, 1994; Radic-Schilling *et al.*, 2023). It encompasses low mountains, plateaus and plains covered by vegetation adapted to drought and wind (Dellafiore, 2018).

At the northern part of the Southern Cone, three key ecoregions are placed at the eastern and western limits of the plains: the Atlantic Forest, the Humid Chaco and the Yungas Forest. The Atlantic Forest (‘Mata Atlântica’), one of the main hotspots of South America, stretches along the eastern coast of Brazil and extends inland towards Argentina and Paraguay. It is dominated by tropical and subtropical broadleaf and semi-deciduous vegetation and includes up to 12 other regions, such as the Alto Paraná Atlantic Forest, the Araucaria Moist Forest and the Serra do Mar Coastal Forest, among many others (Silva, 2018). Also, at the eastern side of the continent, the Humid Chaco spans north-eastern Argentina, central Paraguay and parts of south-western Brazil. This heterogeneous ecosystem combines woodland and savannah (Dellafiore, 2018). On the western side of the plains, the Andean Yungas form a cloud-covered montane forest on the eastern slopes of the Andes, extending from Bolivia to Argentina’s Catamarca province (Brooks, 2018).

Lastly, at temperate latitudes on the western slopes of the Andes, Chile hosts two forested biomes: the Mediterranean and the Valdivian Forests. The Chilean Mediterranean Forest, located in Central Chile, harbours a high proportion of endemic species due to the isolation imposed by the Andes (Hinojosa and Villagrán, 1997). Further south, the Valdivian Forest extends to the limit with Tierra del Fuego. Its constant humid climate favours evergreen vegetation with a dense understorey (Smith, 2018).

### Biogeographical history of the Southern Cone and the Andes

South America’s topographic complexity, with its ecoregions, corridors and barriers shaped by a sequence of geoclimatic since the Oligocene, has greatly influenced the current spatial patterns of South American floristic diversity. These processes are believed to have reshaped the continent’s flora.

The uplift of the Andes stands out as one of the drivers of South America’s floristic complexity. This mountain range provided novel environments for the colonization, radiation and dispersal of lineages, while also acting as a barrier along its length (Van der Hammen and Hooghiemstra, 2000; Luebert and Weigend, 2014; Quiroga *et al.*, 2016; Luebert, 2021; Pérez-Escobar *et al.*, 2022). The uplift of the Andes has been highly diachronous, and many of its impacts were reinforced by climatic events that coincide with the orogenic phases (Hartley, 2003; Strecker *et al.*, 2007). In the Southern Cone, the Andes rose earlier than in the central and northern regions, mainly around the Early–Middle Miocene Boundary (∼16 mya; Blisniuk *et al.*, 2005 ). In the south-central Andes (27–34° S) uplift likely blocked the moist air currents moving westwards, whereas in the central Andes (17–27° S) it had an opposite effect as winds predominantly run eastwards. This orographic barrier to moisture intensified as the uplifting reached its peak, towards the Pliocene (Strecker *et al.*, 2007; Boschman, 2021). By the Late Miocene (∼10.7 mya) most of the southern Andes had nearly reached modern elevations (Boschman, 2021), contrary to the northern areas (Gregory-Wodzicki, 2000), where uplift occurred later in rapid, independent steps at different parts of the cordillera, mostly between the Miocene and Pliocene (∼15–5 mya; Garzione *et al.*, 2006; Lamb, 2015; Boschman, 2021).

During the Middle Miocene, the Mid-Miocene Climate Optimum (17–15 mya; Zachos *et al.*, 2001) was a warming period that increased the sea level, producing marine transgressions at the Southern Cone, forming the Paranean Sea (Hernández *et al.*, 2005; Ortiz-Jaureguizar and Cladera, 2006; Kessous *et al.*, 2020; Luebert *et al.*, 2020) or Paranaense Sea (Murillo-A *et al.*, 2016). These large transgressions created large water bodies between the Andes and the Brazilian eastern shield (Kessous *et al.*, 2020). The sea extended from south-eastern–north-western Argentina to Bolivia (Murillo-A *et al.*, 2016), separating southern from northern South America and altering local bioclimatic and soil conditions.

Towards the end of the Cenozoic, two major dry-vegetation areas acted as barriers separating regions with more mesic conditions: the eastern and the western South American Dry Diagonals. These diagonals fragmented an ancient Palaeocene flora that once extended from coast to coast across the Southern Cone, from subtropical latitudes southwards into Patagonia (Landrum, 1981; Axelrod *et al.*, 1991; Schuettpelz and Pryer, 2009; Palazzesi *et al.*, 2021). As a result, several clades show disjunctions both there and along the Andes (Villagrán and Hinojosa, 1997; Asteraceae, Lörch *et al.*, 2021). The eastern South America Dry Diagonal (eSADD) is an east–west corridor of dry, open vegetation (Ledo and Colli, 2017; Trujillo-Arias *et al.*, 2018; Luebert, 2021; Masa-Iranzo *et al.*, 2021; Bacci *et al.*, 2022) that extends from north-eastern Brazil to north-western Argentina, covering almost of Paraguay and part of Bolivia (Luebert, 2021). It caused the fragmentation between the Amazon and the Atlantic Forests, and between the Atlantic Forest and the Yungas (Barthlott *et al.*, 2005; da Silva *et al.*, 2005; Simon *et al*., 2009; Fiaschi *et al.*, 2016), creating a well-studied disjunction in both animals (Prates *et al.*, 2017; Pirani *et al.*, 2020) and plants (Thode *et al.*, 2019; Masa-Iranzo *et al.*, 2021). Despite this, connectivity between the Andean Yungas and the Atlantic Forest persisted during Pleistocene glacial–interglacial periods, via forest corridors along major river systems (Trujillo-Arias *et al.*, 2017, 2018; Peres *et al.*, 2020). The western South American Dry Diagonal (wSADD; Luebert, 2021) is an extensive arid corridor that extends on the west side of the Andes from Peruvian deserts to the Patagonian Steppe (Hinojosa, 2005; Luebert, 2021). Its southern end lies in the Patagonian Steppe, a biome that became dominant at southern latitudes given the increase in harsh climate conditions caused by several concurring factors. At the same time, the Antarctic Circumpolar Current intensified its effects during the Middle Miocene due to the widening of Drake’s Passage (since Early Oligocene; Cantrill and Poole, 2012; Bacon *et al.*, 2015). The synergic action of a significant pulse of the Patagonian Andean orogeny during the Miocene (∼16 mya) progressively established a rain shadow effect over the eastern flanks of Patagonia (Zachos *et al.*, 2001; Blisniuk *et al.*, 2005; Garreaud *et al.*, 2009; Luebert, 2021). The wSADD was finally consolidated by Pliocene and Quaternary glacial cycles, starting at 7 mya with the Oldest Patagonia Glaciation and ending at 18 kya with the Last Glacial Maximum (Rabassa *et al.*, 2005, 2011).

These geoclimatic episodes ultimately gave rise to distinct disjunction patterns in the Southern Cone. A notable example is the ‘southern Andean–southern Brazilian disjunction’, where species occur disjunctly between the western slopes of the southern Andes (currently Chile) and the southern portion of the Atlantic Forest and the humid Pampa (see Luebert *et al.*, 2020 for examples). This pattern has been linked to vicariance caused by the uplift of the Andes (Landrum, 1981), the Miocene marine transgressions (Kessous *et al.*, 2020; Luebert *et al.*, 2020) and/or the formation of the wSADD (Villagrán and Hinojosa, 1997; Moreira-Muñoz, 2011), as well as to possible dispersal across these barriers (Luebert *et al.*, 2020).

A second reported biogeographical pattern is the ‘south–central Andean disjunction’, which involves lineages distributed in the south-central and southern Andes, as well as in the wetter and milder areas of the central and northern Andes, being absent from the drier zones (Luebert and Weigend, 2014; for example lineages see Lörch *et al.*, 2021). Although less studied than the previous pattern, it has been observed in several Andean groups. It is explained by dispersal corridors that enabled the exchange of cold-tolerant floristic elements, with connectivity periodically disrupted by Quaternary climatic oscillations and the spread of aridity throughout the Andes (Luebert and Weigend, 2014; Lörch *et al.*, 2021). According to Tribble *et al.* (2024), two main spread and colonization pathways may account for this disjunction: (1) north to south, where lineages colonized first from the Northern Hemisphere into the already uplifted northern Andes, and later dispersed to the southern Andes; and (2) south to north, where lineages originated in the older southern Andes and spread northwards, tracking the emergence of new montane habitats (example lineages at Luebert and Weigend, 2014).

### *Carex* as study group in South America

*Carex* (Cyperaceae), with >2000 species, is among the two largest genera within monocots (POWO, 2024). The genus originated in the Late Eocene (∼37 mya), probably in South-East Asia, from where it spread to achieve a nearly cosmopolitan distribution, primarily in temperate–cold climates (Martín-Bravo *et al.*, 2019). About 200 species of the genus are native to South America (Jiménez-Mejías, 2017), though *Carex* taxonomy there is still undergoing major rearrangements (Morales-Alonso *et al.*, 2024*a*). Neotropical *Carex* species mostly occupy areas within temperate–cold climates such as high-latitude (Patagonian) steppes, forests and pampas, as well as mountainous areas in tropical latitudes. South American *Carex* diversification has been shown to result from several independent colonization events from the Northern Hemisphere, primarily Nearctic, western Palaearctic and east Asia regions, demonstrating that the southern hemisphere acted as a sink for the Northern Hemisphere *Carex* diversity (Martín-Bravo *et al.*, 2019; Benítez-Benítez *et al.*, 2021). In spite of the remarkable dispersal ability reported for *Carex* species, the vast majority of the species of the genus lack clear dispersal syndromes and their diaspores are considered to be unspecific (Villaverde *et al.*, 2017).

**Fig. 1.**
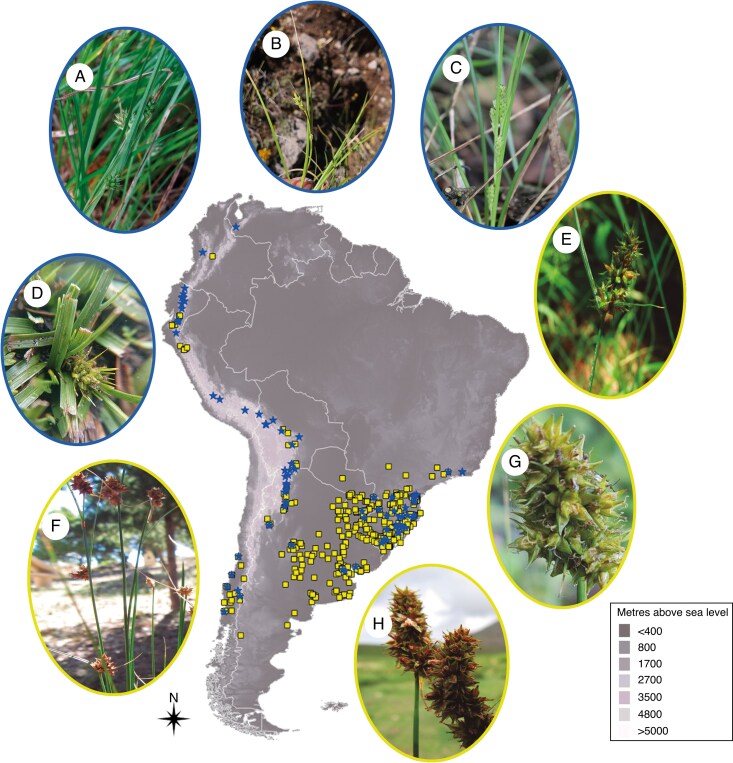
Map showing the distribution of the study group: blue stars for *C. phalaroides* group and yellow squares for sect. *Bracteosellae.* Around the map are displayed representative images of living specimens of the *C. phalaroides* group (A–D), and sect. *Bracteosellae* (E–H). (A) *C. chlorolepis* (Concepcion, Chile). (B) *C. melliza* (Acomayo, Peru). (C) *C. paraguayensis* (Rio Grande do Sul, Brazil). (D) *C. via-montana* (Pichincha, Ecuador). (E) *C. bracteosa* (Concepcion, Chile). (F) *C. fossa* (Buenos Aires, Argentina). (G) *C. guaguarum* (Loja, Ecuador). (H) *C. subdivulsa* (La Rioja, Argentina). Pictures: Paulo Muñoz-Schüler (A, E); Lucely L. Vilca Bustamante (iNaturalist, B); Pedro Jiménez-Mejías (C, G); Ana Morales-Alonso (D); Santiago Martín-Bravo (F, H).

In this study, we explored two taxonomic groups within the genus *Carex* that exhibit similar distribution patterns and ecological adaptations in South America (Fig. 1). These groups are the *Carex phalaroides* group and the section *Bracteosellae*, primarily distributed in the temperate areas of the eastern Southern Cone, with species disjunct through and across the Andes. Each of the two groups is distinguished by their unique morphological characteristics while sharing relatively unspecific ecological preferences and a similar geographical distribution (Tables 1 and 2). Both groups also lack any evident features that may point to particular dispersal modes (Jiménez-Mejías *et al.*, 2021; Morales-Alonso *et al.*, 2024*b*).

**Table 1.** Summary of the distribution and habitat of the six species that compose the *C. phalaroides* group.

Species	Distribution	Habitat
*C. chlorolepis*	Central Chile and Juan Fernández archipelago	Dry shaded soils, from anthropized environments to open lauroid Valdivian forest; 20–600 m
*C. gibertii*	NE Argentina (Buenos Aires province), Uruguay and SE Brazil (Paraná, Rio Grande do Sul)	Dry soil, in open habitats such as pampas or scrubland; 0–400 m
*C. melliza*	NW Argentina, Bolivia, S and central Peru	Mesic soils, in open habitats in high-altitude shrublands or open Yungas montane forest; 1600–3350 m
*C. paraguayensis*	SE Brazil (Paraná, Santa Catarina, Rio Grande do Sul), NE Argentina (Misiones), Paraguay	Wet to mesic soils, on shaded understorey in the Atlantic Forest broadleaf and Araucaria Moist Forests; 550–1700 m
*C. phalaroides*	NE Argentina, Uruguay, and SE Brazil (Paraná, Santa Catarina, Rio Grande do Sul)	Mesic to moist soils on shaded to open habitats, from grasslands to forest understorey; 150–1700 m
*C. via-montana*	Tropical Andes from N Peru, to Ecuador, Colombia and Venezuela, disjunct in Guatemala	Mesic soils on open grass and shrub montane formations; 2800–4100 m

Distribution and habitat information is extracted from the taxonomic treatment published in Morales-Alonso *et al.* (2024*b*).

**Table 2.** Summary of the distribution and habitat of the eleven species that compose *Carex* sect. *Bracteosellae*.

Species	Distribution	Habitat
*C. bonariensis*	NE and E Argentina, marginally in W Argentina, Uruguay, S Brazil. Introduced in Chile	Open habitats; meadows and lake banks; 0–200 m
*C. bracteosa*	Central Chile	Open habitats; wet meadows and seasonally flooded environments; 0–800 m
*C. feddeana*	NE and E Argentina, Uruguay, S Brazil, and marginally in Bolivia	Open habitats; wet meadows and swamps; 0–1000 m
*C. fossa*	NE and E Argentina, Uruguay and S Brazil	Open habitats; grassy fields, pasture lands, slimy soils at riverbanks, and roadside verges; 0–450 m
*C. giovanniana*	W Argentina, and S Bolivia, in the central Andes	Open habitats and partly shaded soils; meadows and forest clearings in Yungas forests; 1800–2650 m
*C. guaguarum*	Tropical Andes from N Peru, to Ecuador, and marginally in Colombia	Open habitats and partly shaded soils; meadows and open grass clearings; 2800–4100 m
*C. pedicularis*	NE and E Argentina, marginally in W Argentina.	Open habitats; meadows in forest edges; 0–100 m
*C. rupicola*	E Argentina (Sierra de la Ventana)	Open habitats; dry sites on rocky cliffs or ledges; 0–500 m
*C. sororia*	NE and E Argentina, Uruguay and S Brazil	Open habitats; wet meadows; 0–1500 m
*C. subdivulsa*	W Argentina (Sierra de Famatina)	Open habitats; moist meadows; 2500–2800 m
*C. uruguensis*	E Argentina, Uruguay and S Brazil	Open habitats; wet meadows; 0–500 m

Distribution and habitat information is extracted from the taxonomic treatments published in Jiménez-Mejías *et al.* (2021) and Muñoz-Schüler *et al.* (Accepted).

The *C. phalaroides* group is a taxonomic complex within subgenus *Psyllophorae*, section *Junciformes*, the only representation of the subgenus in South America. According to Morales-Alonso *et al.* (2024*b*) it comprises six taxa (Table 1, Fig. 1). The origin and diversification of sect. *Junciformes* mostly happened in Patagonia, where the group has retained an overall conserved temperate–cold niche (Benítez-Benítez *et al.*, 2021). In contrast to most other species of the section, the *C. phalaroides* group shows a clear niche shift and has expanded into temperate and subtropical regions. It occurs along the Atlantic coast of South America, being also disjunct in Chile, and extending northwards into tropical latitudes along the Andes. Species within the *C. phalaroides* group have adapted remarkably to a variety of habitats (Morales-Alonso *et al.*, 2024*b*): subtropical humid forests (such as the Atlantic Forest or the Andean Yungas), temperate pampas, temperate forests such as the Valdivian Forest, and high-altitude Andean grasslands (puna and paramo) (Dinerstein *et al.*, 2017).

*Carex* sect. *Bracteosellae* is the only section of subgenus *Vignea* that is primarily diversified in the South American continent. It comprises 11 species (Table 2, Fig. 1) (Jiménez-Mejías *et al*., in press). The section is primarily centred in the Atlantic region of the Southern Cone, with a few species extending to the northern Andes and Chile. While most species occupy wet to mesic habitats such as meadows, *C. rupicola* has apparently adapted to xeric conditions (Jiménez-Mejías *et al.*, 2021). Most species inhabit the Pampa and Alto Paraná ecoregions; however, *C. giovanniana* and *C. subdivulsa* are restricted to meadows in the Yungas ecoregion, and *C. bonariensis* can occasionally be found in such habitats. The recently described *C. guaguarum* is the only species that reaches the northern Andes, and *C. bracteosa* is restricted to lowland open habitats in central Chile. Additionally, *C. bonariensis* has been reported as introduced in Chile (Muñoz-Schüler *et al.*, Accepted).

The similar distribution patterns of the *C. phalaroides* group and sect. *Bracteosellae* across South America provide an ideal scenario to explore key evolutionary questions at a broad geographical scale and thus consider macroecological requirements. Do these groups show parallel synchronic evolutionary histories? Are these patterns linked to the geoclimatic processes that have shaped the current environmental landscape of the Southern Cone; and if so, what are those events? To answer these questions, we conducted phylogenomic, divergence time estimation and biogeographical and bioclimatic niche analyses. Our approach helps to shed light on the understanding of these underexplored systems.

## MATERIALS AND METHODS

### Sampling and DNA extraction

For the *C. phalaroides* group, we included ten samples: one of *C. chlorolepis*, one of *C. gibertii*, three of *C. melliza*, two of *C. paraguayensis*, two of *C. phalaroides* and one of *C. via-montana* (Supplementary Data Table S1). We tested the phylogenetic position of our in-group by including representative samples from all the major clades within subg. *Psyllophorae* (Roalson *et al.*, 2021): (1) *C. curvula* and *C. baldensis*; (2) *C. oedipostyla* from sect. *Psyllophorae*; (3) *C. schimperiana* from sect. *Schoenoxiphium*; and (4) *C. camptoglochin*, *C. acicularis* and *C. vallis-pulchrae* subsp. *vallis-pulchrae* (Aciculares Clade) from sect. *Junciformes* together with *C. patagonica* (Junciformes clade). For sect. *Bracteosellae* (Supplementary Data Table S1) we included 13 samples: one sample for each species in the section and two for *C. bracteosa*. The out-group selection included one representative from each of three sister sections within subg. *Vignea* (Roalson *et al.*, 2021): *C. disperma* from sect. *Dispermae*; *C. maritima* from the Disticha clade; and *C. gibba* from sect. *Gibbae*. Almost all samples included were newly sequenced for this study. The only exceptions are seven out-group samples, which were extracted from GenBank and already included in Léveillé-Bourret *et al.* (2018) and Villaverde *et al.* (2020) (Supplementary Data Table S1).

Genomic DNA extraction was performed using a modified CTAB procedure (Doyle and Doyle, 1987) and subsequently quantified to ensure optimal concentrations for genomic sequencing (≥50 ng µL^−1^). Library preparation and sequencing were carried out by Arbor Biosciences (MI, USA) for all the samples except for *C. guaguarum* and both *C. bracteosa* samples. Due to their fresh condition, these three samples were sonicated in a Bioruptor Pico Ultrasonicator to achieve a fragment size of 550 bp. Libraries were then prepared with the NEBNext Ultra II DNA Library Prep Kit for Illumina (New England Biolabs, MA, USA) and subsequently quality-checked using a Qubit 2.0 Fluorometer. Once the samples were indexed, they were pooled in equimolar amounts (500–700 ng) into groups of ∼15 samples. Enrichment was performed using the Cyperaceae-specific baits kit (Villaverde *et al.*, 2020), followed by hybridization according to the myBaits kit protocol from Arbor Biosciences. DNA sequencing was conducted by Arbor Biosciences on an Illumina HiSeq NovaSeqX 150 PE platform. The trimmed paired reads are available in GenBank, Bioproject PRJNA1225250.

### Assembly of nuclear data and phylogenomic analyses

Raw reads were quality-trimmed using Trimmomatic (Bolger *et al.*, 2014) with default parameters and checked before and after with FastQC (Andrews, 2010). The paired sequences were assembled using the HybPiper v.2.3. pipeline (Johnson *et al.*, 2016) applying the BWA option and the Cyperaceae-specific sequence references (Villaverde *et al.*, 2020). The pipeline implements SPAdes (Bankevich *et al.*, 2012) to assemble the reads into contigs by mapping them against the reference sequences. HybPiper statistics were obtained with Samtools v.1.21 (Danecek *et al.*, 2021). Exon matrices were aligned with MAFFT v.7.222 (Katoh and Standley, 2013) with the ‘auto’ function. Aligned gene matrices were used for both concatenation and coalescence approaches. AMAS (Borowiec, 2016) was employed to concatenate aligned gene matrices and to obtain summary statistics of exon matrices. For the concatenated approach, we constructed an exon matrix by combining single gene matrices using AMAS. Then, IQ-TREE v.2.3.6 (Minh *et al.*, 2020*a*) was used to obtain the species tree, applying an ultrafast bootstrap option (Hoang *et al.*, 2018) with a prior automatic model selection using ModelFinder (Kalyaanamoorthy *et al.*, 2017). Branch support for the concatenated approach was provided using the Shimodaira–Hasegawa approximate likelihood ratio test (SH-aLRT) and ultrafast bootstrap (UFBoot), both set to default with ‘-UFBoot 1000’ and ‘-alrt 1000’. Gene trees for the coalescence approach were obtained using RAxML (Stamatakis, 2014) with default settings, applying the GTRCAT model and 200 fast bootstraps. We inferred the species coalescence tree with ASTRAL III v.5.6.1 (Zhang *et al.*, 2017), as input we used the individual gene trees from RAxML and support was provided by local posterior probability (LPP). Additionally, two measures of genealogical concordance were calculated using IQ-TREE (Minh *et al.*, 2020*b*) for quantifying genealogical concordance in our phylogenomic dataset: the site concordance factor (sCF) and the gene concordance factor (gCF). Low values of concordance factors were considered when gCF <10 % and sCF <33 %, following Minh *et al.* (2020*b*). Alternative quartet topologies were assessed using ASTRAL III v.5.6.1 to determine the percentage of quartets in gene trees that agreed with a branch, thereby measuring the extent of gene tree conflict around that branch. These quartets were then plotted using the treeio (Wang *et al.*, 2020) and ggtree (Yu *et al.*, 2018) packages in RStudio v.1.4.1717, after rooting with the APE R package (Paradis, *et al.*, 2004).

### Divergence time estimation

As stated by Villaverde *et al.* (2023), divergence time estimation using a Bayesian approach can be a computationally inefficient task for different reasons (large size of datasets, incongruence among tree topologies, etc.). For this reason, we employed the ‘gene shopping’ approach to create a concatenated matrix using only those aligned exons with the highest quartet values, indicating they were equally resolved in both the IQ-TREE and the individual gene tree topologies obtained with RAxML. Using this procedure, we created a 20-locus matrix for each study group: a 39 588-bp matrix for the *C. phalaroides* group and a 38 308-bp matrix for sect. *Bracteosellae*. Both matrices were used as input for IQ-TREE in order to check if the resultant topologies matched the ones recovered by the aforementioned IQ-TREE analyses. For the *C. phalaroides* group analysis, we pruned the tree leaving only one tip per taxon of sect. *Junciformes* species. The crown node of sect. *Junciformes* was calibrated using a secondary calibration point, obtained from a broader divergence time estimation analysis made by Benítez-Benítez *et al.* (2021) (mean = 16.05 mya, *σ* = 2, 95 % highest probability density = 12.86–19.17). For sect. *Bracteosellae* we pruned the tree, leaving only one tip per taxon and we used two calibration points: a fossil calibration for the crown node of subgenus *Vignea* (23–16 mya, *σ* = 1; Jiménez-Mejías *et al.*, 2016) and a secondary calibration point for sect. *Bracteosellae* (mean = 6.22, *σ* = 2) from Martín-Bravo *et al.* (2019). Following Villaverde *et al.* (2023), both analyses were carried out in BEAST v.1.10.4 (Suchard *et al.*, 2018) using the GTR + G substitution model, the uncorrelated relaxed log-normal clock model (Drummond *et al.*, 2006), and the birth–death model with incomplete sampling (Stadler, 2009). As the initial starting trees to inform the process, we used the ASTRAL topologies dated in treePL (Smith and O’Meara, 2012). In both analyses, we ran the MCMC chain for 200 million generations, sampling every 20 000 generations and discarding the first 20 million states as burn-in. We ensured that all analysis parameters achieved an effective sample size (ESS) using Tracer v.1.7.1 (Rambaut *et al.*, 2018). We generated the maximum clade credibility (MCC) tree using TreeAnnotator v.1.10 (Drummond *et al.*, 2006), which summarizes clade support as posterior probability values.

### Ancestral area reconstruction

For ancestral area reconstruction we used the BioGeoBEARS R package (Matzke, 2018). Dispersal extinction-cladogenesis (DEC), dispersal-vicariance analysis (DIVA-like) and a likelihood interpretation of BayArea (BAYAREA-like; Matzke, 2014) models were tested without the +J parameter, addressing concerns raised by Ree and Sanmartín (2018). The fit for each model was evaluated using the corrected Akaike information criterion (AICc). Ancestral area reconstruction analysis was performed using the previously obtained chronograms, pruned to retain only one tip per species in both trees (see Results) and one species per out-group section/clade. A biogeographical matrix was constructed by coding the South American ecoregions where the species of sect. *Junciformes* and sect. *Bracteosellae* are present, based on the georeferenced dataset provided by Sanz-Arnal *et al.* (2025a, 2025b). The codification of areas for the in-groups was carefully compared with existing regionalization proposals for the continent (Martínez *et al.*, 2011; Morrone *et al.*, 2022; Morrone and Ebach, 2022). Six areas were defined and coded: (1) A, Atlantic region, comprising occurrences from the Atlantic Forests (*Araucaria* moist forest, Alto Paraná Atlantic Forest) as well as scattered marginal presences in the neighbouring Humid Chaco; (2) P, Pampa, including Humid Pampa, Espinal, Uruguayan Savanna, Mesopotamia and those marginal occurrences from the neighbouring Arid Chaco; (3) C, central Andes, including southern Andean, Bolivian and Peruvian Yungas and the marginal occurrences from the central wet Puna; (4) W, western flanks of the southern Andes (Chile, including the Mediterranean and Valdivian forests); (5) N, northern Andes, including Andean regions north of the Huancabamba depression; and (6) O, others, including areas outside of the geographical regions of interest (i.e. areas occupied by the out-groups) (Supplementary Data Table S2). Additionally, a simple dispersal multipliers matrix was added to the analysis where adjacent areas were given a score of 1 and non-adjacent areas were given a score of 0.5, limiting the dispersal movements considered in the analyses. A score of 0.25 was given to areas for which dispersal was less probable due to topographic barriers (N to W and W to C), as well as all areas to O.

#### Estimation of number, type and directionality of biogeographical events

We evaluated the type and number of biogeographical events using the biogeographical stochastic mapping (BSM) implemented in BioGeoBEARS (Dupin *et al.*, 2016; Matzke, 2016). This method aids in building models of trait evolution and estimating historical changes along branches (Nielsen, 2002; Huelsenbeck *et al.*, 2003), including both anagenetic and cladogenetic events (Dupin *et al.*, 2016). The best models for both the *C. phalaroides* group and sect. *Bracteosellae* were analysed, generating simulated histories with event times and locations. Following Dupin *et al.* (2016), event frequencies were estimated by calculating the mean and standard deviation of event counts from 100 BSMs.

Stochastic character mapping (Freyman and Höhna, 2019) was conducted for both groups using RevBayes (Höhna *et al.*, 2016) to support the directionality estimations of geographical range changes along branches made with BSM. This software also allowed us to estimate uncertainty in anagenetic parameters and ancestral nodal ranges by calculating marginal posterior probabilities (Landis *et al.*, 2018; Lavor *et al.*, 2019; Villaverde *et al.*, 2023) using the DEC model (Landis *et al.*, 2018). We used the same pruned ASTRAL trees, biogeographical regions and occurrence datasets as in the previous BioGeoBEARS analyses. A simple RevBayes phylogenetic analysis was run using default priors for 10 000 generations, sampling every tenth generation and dividing the phylogeny into 500 time slices. Following Villaverde *et al.* (2023), we summarized the results into a tree with marginal probabilities for ancestral nodal ranges and branch-mapped dispersal events. All outputs were visualized using the R package RevGadgets v.1.0 (Tribble *et al.*, 2022).

### Bioclimatic niche analyses

#### Bioclimatic preferences

Occurrences were obtained from the georeferenced dataset by Sanz-Arnal *et al*. (2025*a*, 2025*b*) (Supplementary Data Table S3). We retrieved 19 bioclimatic variables from WorldClim2 (Fick and Hijmans, 2017) and retained five variables for both groups based on the correlation matrix (selecting one variable per clade branch longitude <0.7), and the variance inflation factor (0.5 < VIF < 5): (1) Bio 1, annual mean temperature; (2) Bio 4, temperature seasonality; (3) Bio 12, annual precipitation; (4) Bio 15, precipitation seasonality; (5) Bio 18, precipitation of warmest quarter. These bioclimatic variables were plotted as density curves per taxon to visualize species responses to each variable and their overlap. Additionally, a PCA was performed with the retained bioclimatic variables to visualize the environmental space using the ggplot2 R package (Wickham, 2006).

#### Ancestral niche reconstruction

Ancestral state reconstructions of the lineages’ ecological preferences were conducted using the phytools R package (Revell, 2012), employing the chronogram and the mean value of the retained bioclimatic variables by species (see Results). To provide an integrative representation of the bioclimatic niche, we plotted the mean values of PC1 and PC2 from the PCA for each taxon. Before the definitive analyses, we tested two evolutionary models for continuous traits, Brownian motion (BM) and Ornstein–Uhlenbeck (OU) and selected the best fit using the AICc as implemented in the GEIGER R package (Harmon *et al.*, 2008).

## RESULTS

### Phylogenomic analyses

Of the 554 loci matrices retrieved with the Cyperaceae-specific baits, we discarded those not recovered for several species and the ones identified as paralogues, leaving a set of 465 locus matrices for the *C. phalaroides* group and 520 for sect. *Bracteosellae*. All the HybPiper statistics, sequence lengths and gene matrix summaries for both lineages can be found in Supplementary Data Tables S4–S7. The phylogenies retrieved for the *C. phalaroides* group and sect. *Bracteosellae* showed both study groups as monophyletic, with identical internal topologies and strong support using both concatenated and coalescence approaches (SH-aLRT = 100, UFBoot = 100; LPP = 1; Supplementary Data Fig. S1). Minor topological differences affecting the out-group were observed only in the *C. phalaroides* group tree. *Carex paraguayensis* (Clade A) was sister to the rest of the *C. phalaroides* group. The rest of the group was subsequently divided in the other two clades: Clade B with *C. gibertii* + *C. melliza*, and Clade C with *C. chlorolepis* + *C. phalaroides* + *C. via-montana* (Supplementary Data Fig. S1A). The sect. *Bracteosellae* was divided into two main clades: Clade A, grouping *C. giovanniana* + *C. divulsa* and *C. rupicola* + *C. pedicularis* + *C. fossa*, and Clade B, grouping both samples of *C. feddeana* in one clade and *C. sororia* + *C. uruguensis* + *C. bonariensis* + *C. guaguarum* + *C. bracteosa* in another (Supplementary Data Fig. S1B).

High gCF and moderate sCF values were again recovered for the *C. phalaroides* (gCF = 82.64 %; sCF = 38.36 %) clade. Clade A obtained the highest concordance factor values (gCF = 90.65 %; sCF = 89.35 %). Clade B reached lower values (gCF = 17.35 %; sCF =37.57 %) compared with Clade C (gCF = 28.2 %; sCF = 52.71 %). For the rest of the clades, we obtained high values of concordance factors for almost every branch except for the *C. camptoglochin* one (gCF = 14.79 %; sCF = 27.52 %), with only 25 genes and 89.28 sites supporting that branch. Section *Bracteosellae* also retrieved high gene and site concordance. Clade A (Supplementary Data Fig. S1B) reached higher concordance factors (gCF = 36.28 %; sCF = 59.37 %) than Clade B (gCF = 19 %; sCF = 35.15 %; Supplementary Data Fig. S1B). Within Clade A, all gCFs had values around 70 % while sCF values were between 62 and 72 %. Concordance factors within Clade B were lower for every node except for *C. feddeana* (gCF = 69.67 %; sCF = 79.94 %) when compared with Clade A. The *C. sororia* branch reached the lowest gCF values (28.21 %). The second lowest gCF value was retrieved in the *C. uruguensis* node (gCF = 28.02 %; sCF = 44.33 %) and the third lowest gCF value was found in the *C. bracteosa* node (gCF = 34.75 %; sCF = 49.64 %).

Regarding the quantification of quartet distance from each gene tree to the concatenated tree, the reference topology of the *C. phalaroides* group was supported by 93.5 % of the single-locus trees (Supplementary Data Fig. S1A). For Clades A, B and C the reference topology was retrieved by 93.3, 38.9 and 55.8 % of the single-locus trees, respectively. Within Clade C, the *C. phalaroides* + *C. via-montana* clade retrieved the reference topology in 46.4 % of the trees, while the first alternative topology was recovered in 26 % of the single-locus trees and other alternatives in 27.6 % of the single-locus trees.

For sect. *Bracteosellae* (Supplementary Data Fig. S1B), the reference topology was supported by 60 % of the single-locus trees in Clade A and by 40 % Clade B. Within Clade A, *C. giovanniana* + *C. subdivulsa* was retrieved by 70 % of the single-locus trees, and the *C. rupicola* + *C. pediculari*s + *C. fossa* clade was supported by 75 % of single-locus trees. In Clade B, the *C. feddeana* clade was retrieved by 81 % of the single-locus trees, while the reference topology of the rest of the clades was supported by 53 % (*C. sororia*), 47 % (*C. uruguensis*), 78 % (*C. bonariensis*) and 55 % (*C. guaguarum* + *C. bonarensis* + *C. bracteosa* clade) of the single-locus trees.

### Divergence time

The tree topology obtained with the divergence time estimation analysis of the *C. phalaroides* group with BEAST was identical to the one obtained with ASTRAL III (Supplementary Data Fig. S2A). However, it yielded slightly different results compared with those obtained by Benítez-Benítez *et al.* (2021), with overall ages older. The divergence time of the *C. phalaroides* group stem node was inferred at 14.9 mya during the Middle Miocene (95 % highest posterior density (HPD) = 10.82–18.81). The initial diversification of the *C. phalaroides* group was estimated at 8.88 mya, also in the Middle Miocene (95 % HPD = 3.65–15.05), coinciding with the divergence of the *C. paraguayensis* lineage. During the Late Miocene the divergence of Clades B and C took place (6.31 mya, 95 % HPD = 1.75–13.72), followed by the separation of *C. gibertii* from *C. melliza* (3.50 mya, 95 % HPD = 0.24–7.67). During the Early Pliocene, *C. chlorolepis* diverged from the *C. phalaroides* and *C. via-montana* (4.15 mya, 95 % HPD = 0.55–10.63), and the latter two species diverged almost during the Late Pliocene (3.50 mya, 95 % HPD = 0.24–7.67).

For sect. *Bracteosellae* (Supplementary Data Fig. S2B), its stem node started to diverge from the Late Miocene (6.69 mya, 95 % HPD = 3.8–10.1), with further diversification occurring mainly throughout the Pliocene. At 5.89 mya (95 % HPD = 2.8–9.2), the divergence of the *C. feddeana* lineage marked the beginning of Clade B diversification. This was followed by the divergence of *C. sororia* (5.11 mya, 95 % HPD = 1.9–8.1) and *C. uruguensis* (4.26 mya, 95 % HPD = 1.3–7.2) and the diversification of *C. bonariensis* (2.72 mya, 95 % HPD = 0.6–4.9). This last clade diversified during the Early Pleistocene, with an early divergence of the *C. bonariensis* lineage and the split between *C. bracteosa* and *C. guaguarum* at 1.47 mya (95 % HPD = 0.2–3.1). A splitting event in the Early Pliocene (4.75 mya, 95 % HPD = 1.7–8.1) between the two subclades within Clade A (*C. giovanniana* + *C. subdivulsa* and *C. rupicola* + *C. pedicularis* + *C. fossa*) marks the initial diversification of this group. *Carex rupicola* diverged at 2.56 mya (95 % HPD = 0.5–5.2) from a clade formed by *C. pedicularis* and *C. fossa.* These two species represent the latest divergence within the evolution of sect. *Bracteosellae*, occurring during the Early Pleistocene at 1.25 mya (95 % HPD = 0.1–3.2). Finally, the lineages of *C. giovanniana* and *C. subdivulsa* diverged from each other at 1.65 mya (95 % HPD = 0.03–4.2).

### Ancestral area reconstruction

For the *C. phalaroides* group, the DIVA-like model was selected as the best fit for the group analysis (AICc = 47.8) as it yielded a lower AICc than both BAYAREA-like and DEC models (AICc = 51.7 and 52.6, respectively). However, the DEC model reconstruction cannot be entirely ruled out, as the AICc difference from the DIVA-like model was not significant (Burnham *et al.*, 2011). In any case, as DIVA-like and DEC models recovered the same ancestral area for each lineage considered, for the sake of simplicity we will describe only the results from the DIVA-like model. All models retrieved a high proportion of uncertainty. The DIVA-like model identified the Atlantic region and Pampa (P and A, respectively) as the ancestral area for the entire group (30.7 %). Additionally, the Atlantic region alone was retrieved with 18 % probability. Regarding the rest of the nodes: (1) the ancestral area for Clade B + Clade C with 18.6 % probability was retrieved as the A + P combination and 15.2 % probability for just the Atlantic region; (2) the most probable ancestral range area estimated for Clade B corresponds to the Atlantic region + central Andes (A + C) combination with 31.5 % and P + C with 29.4 %; (3) the most probable area of distribution for the common ancestor of species in Clade C corresponds to Atlantic + Chile areas (A + W, 17.7 %), 16.1 % for P + W, and 17.2 % for the Chile + north Andes combination of areas (W + N); and (4) the *C. via-montana–C. phalaroides* divergence node retrieved 42.4 % probability for A + N shared areas and 39.9 % for the P + N combination. Despite the uncertainty recovered by the DIVA-like analysis, according to these results the group probably had an A + P origin with a quick spread to the W and C areas and a final expansion towards the Andes (N).

For sect. *Bracteosellae* the best fit model was DIVA-like (AICc = 78.75), which yielded a comparatively lower AICc value than the competing models (DEC and BAYAREA-like; AICc = 82.03 and 86.26, respectively). Ancestral range reconstruction under the selected model recovered a combination of the Atlantic region, the Pampa and the central Andes as the most probable ancestral range for the group (70 %). Clade A’s ancestral range was most probably located between the Pampa and the central Andes (99.7 %). Internal nodes recovered ancestral areas in the central Andes for the *C. giovanniana–C. subdivulsa* clade (∼100 %) and the Pampa for the diverging node (99.6 %). The Atlantic region was retrieved as the most probable ancestral area for Clade B (76.3 %) as well as most of its internal nodes. This suggests a long-term residence of the group within this area. According to our results, *C. bonariensis* probably originated in a widespread range comprising the Atlantic region, the Pampa and the western Andes (61.7 %), suggesting a range expansion towards the Andes. This is also associated with the ancestral range retrieved for the *C. guaguarum–C. bracteosa* clade, which resulted in a combination of the northern Andes and the western flanks of the southern Andes (∼100 %).

### Number, type and directionality estimation of biogeographical events

The summary of BSMs indicated that most of the biogeographical events within the *C. phalaroides* group species were anagenetic dispersions (64.8 %), followed by within-area speciation (17.7 %) and vicariance (16.47 %) (Table 3). Sympatric events would have occurred only within the A + O, followed by several range expansion events. The cladogenetic vicariance events are inferred in the most external nodes, involving the fragmentation of the combination of areas that include the Atlantic region (A + C, A + N and A + W).

**Table 3.** Summary of BSM (100 runs) counts using the DIVA-like model for *C. phalaroides* group and DIVA-like model for sect. *Bracteosellae*.

		*C. phalaroides* group		sect. *Bracteosellae*	
Mode	Type	Mean (s.d.)	%	Mean (s.d.)	%
Anagenetic	Range expansion (*d*)	11.04 (1.56)	64.8	11.14 (0.78)	46.1
	Range contraction (*e*)	0		0	
Cladogenetic	Sympatry (*y*)	1.37 (1.1)	8.03	7.72 (1.03)	31.9
	Speciation – subset	0		0	
	Vicariance	4.63 (1.1)	27.2	5.28 (1.03)	21.9
	Founder event (*j*)	0		0	
Total		17.04 (1.568)		24.14 (0.78)	

All the biogeographical processes contemplated in the analysis are here indicated with their respective parameter in parentheses.

Mean numbers of the different types of event estimated are shown here along with standard deviations.

Similarly, for sect. *Bracteosellae*, the most common biogeographical events estimated by BSMs were anagenetic dispersions (46.1 %), followed by sympatric speciation (31.98 %) and vicariance (21.9 %) (Table 3). Narrow sympatric events were estimated to have occurred mostly within the Pampa region. Cladogenetic vicariance events mostly accounted for the range between the Pampa and central Andes regions.

**Fig. 2.**
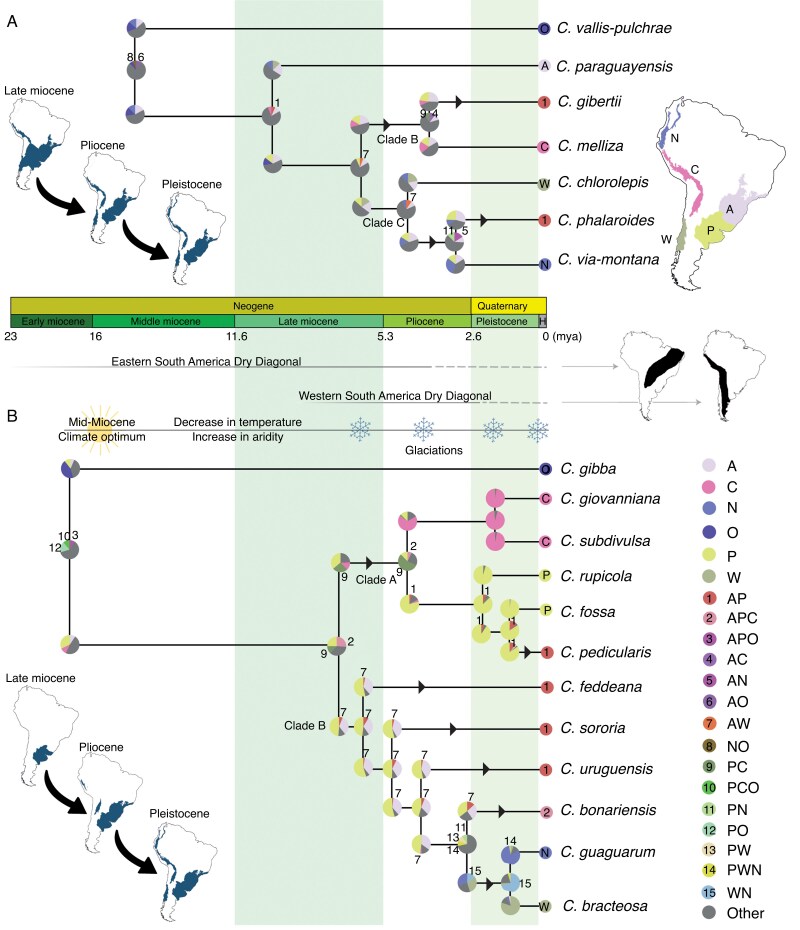
Geographical range evolution of (A) the *C. phalaroides* group and (B) sect. *Bracteosellae*. On the right is a map indicating the five areas of distribution used in every biogeographical analysis. Colour codes for widespread ranges: A, Atlantic region; P, P& C, central Andes; W, Chile; N, northern Andes; O, other areas. Combinations of areas are displayed with the corresponding number to facilitate interpretation within the phylogenies. Arrowheads represent the estimated moment of the anagenetic dispersal event revealed by the stochastic mapping analysis performed in RevBayes. Under each phylogeny are display map figures summarizing the main vicariance and dispersal events in the history of both groups. Under the time bar, the estimated development of the eSADD and wSADD are marked with lines and so they are climatic events relevant to this study, as the Mid-Miocene Climate Optimum (17–15 mya) and the cycles of Pliocene–Quaternary glaciations (7 mya–18 kya).

The ancestral area reconstruction using RevBayes (Fig. 2A) yielded similar values to those obtained with the BioGeoBEARS analysis for the *C. phalaroides* group. As in the previous analysis, uncertainty within the proportions for the ancestral ranges was high in all deep nodes, despite all branches receiving maximum posterior probability support (PP = 1). The ancestral range estimation tree recovered the same areas with higher proportions than the BioGeoBEARS DIVA-like analysis. However, some exceptions were found. The ancestral area of the whole group was here recovered with similar probabilities for the A, P and W areas, reinforcing the interpretation of a wide origin area (Fig. 2A). Chile was also found as one of the three probable areas for the Clade B + Clade C node, and this analysis did not recover A as one of the most probable areas for the Clade C divergence node. The branch-mapped dispersal events tree (Supplementary Data Fig. S3A) retrieved the Atlantic as the ancestral area for the group, instead of the A + P combination of areas, and did not recover the Atlantic + Chile combination in the Clade C node. The rest of the nodes supported the biogeographical events retrieved in the BSM analysis, with a high proportion of anagenetic events, up to five major events recovered, symbolized by a change of area during the species branch in Supplementary Data Fig. S3A, followed by vicariance in most cases.

The ancestral range reconstruction using RevBayes (Fig. 2B) estimated an uncertain area of origin for sect. *Bracteosellae*, with no region showing high predominance. However, the Clade A + B divergence node obtained an A + P + C combination of areas, reinforcing the interpretation of a widespread range. For Clade A, ancestral areas were recovered the same by the DIVA-like analysis but with generally lower proportions. Ancestral area reconstruction for Clade B recovered the Pampa area as the most probable for all the internal nodes except for *C. bonariensis*, which contrasted with the results obtained in the DIVA-like analysis. The branch-mapped dispersal events tree retrieved for sect. *Bracteosellae* (Supplementary Data Fig. S3B) agreed with BioGeoBEARS analyses in the areas and proportion of speciation events recovered for each species, with a higher proportion of cladogenetic events (*C. bracteosa*, *C. guaguarum*, *C. fossa*, *C. rupicola*, *C. subdivulsa* and *C. giovanniana*) than anagenetic.

### Bioclimatic niche and ancestral niche reconstruction

Principal component analysis performed with the mean values of the retained bioclimatic variables for the occurrences of the *C. phalaroides* group accounted for 80 % of the variance over the first two principal components (PCs) and 90 % on the first three (PC1, 54 %; PC2, 26.3 %; and PC3, 10.6 %; Supplementary Data Table S8). For sect. *Bracteosellae*, the percentage of variance obtained was 88.5 % in the first three components (PC1, 49 %; PC2, 24.2 %; and PC3, 15.3 %; Supplementary Data Table S8). PCAs displayed a trend for precipitation variables along the first PC, while on the second component temperature variables were the ones with more importance (Supplementary Data Table S8). The scatter plot of the two first PCs plotted by species (Supplementary Data Fig. S4A) depicted a separation of two large clusters, one composed only of the eastern South America species (*C. paraguayensis*, *C. phalaroides* and *C. gibertii*) and another with individuals of western South America (*C. chlorolepis*, *C. melliza* and *C. via-montana*). However, some occurrences marginally overlap with the other cluster; this is the case for *C. chlorolepis* with the eastern cluster, and *C. gibertii* and *C. phalaroides* with the western cluster. Additionally, *C. via-montana* displays the clearest separation of clusters in the plot.

For sect. *Bracteosellae*, no clear structure could be seen in the scatter plot of the first two PCs (Supplementary Data Fig. S4B), as species with widespread distribution overlapped with each other (*C. bonariensis*, *C. feddeana*, *C. fossa*, *C. pedicularis*, *C. sororia*) and with more geographically restricted species (*C. rupicola*, *C. uruguensis*). Central Andean species (*C. giovanniana*, *C. subdivulsa*) formed a loose cluster along *C. bracteosa*, showing marginal overlap with some widespread species (*C. bonariensis*, *C. feddeana*). The clearest clustering separation was depicted by *C. guaguarum*.

The response curves of the five bioclimatic variables (Supplementary Data Fig. S5A) show significant overlaps among taxa, except for *C. chlorolepis* in relation to temperature variables. For the precipitation variables Bio 12 and Bio 18, there is a slight separation between eastern and western taxa, with the western taxa *C. melliza* and *C. via-montana* showing lower precipitation values. The precipitation variable Bio 15 reveals two well-differentiated groups: *C. melliza* + *C. via-montana* form one group with higher precipitation seasonality values, while the remaining species constitute another group with lower values for this variable. Regarding temperature variables, all species exhibit a clear overlap except for *C. chlorolepis*, which shows lower values for both Bio 1 (annual temperature) and Bio 4 (temperature seasonality) compared with the other species, this difference being more pronounced for Bio 4. Additionally, *C. via-montana* displays a bimodal distribution for temperature seasonality.

A widespread overlap of bioclimatic variables was observed for sect. *Bracteosellae* (Supplementary Data Fig. S5B), except for *C. rupicola*, which segregated from the other species in all variables except Bio 1. For temperature seasonality (Bio 4), *C. guaguarum* and *C. rupicola* segregated at the lowest and highest values, respectively.

**Fig. 3.**
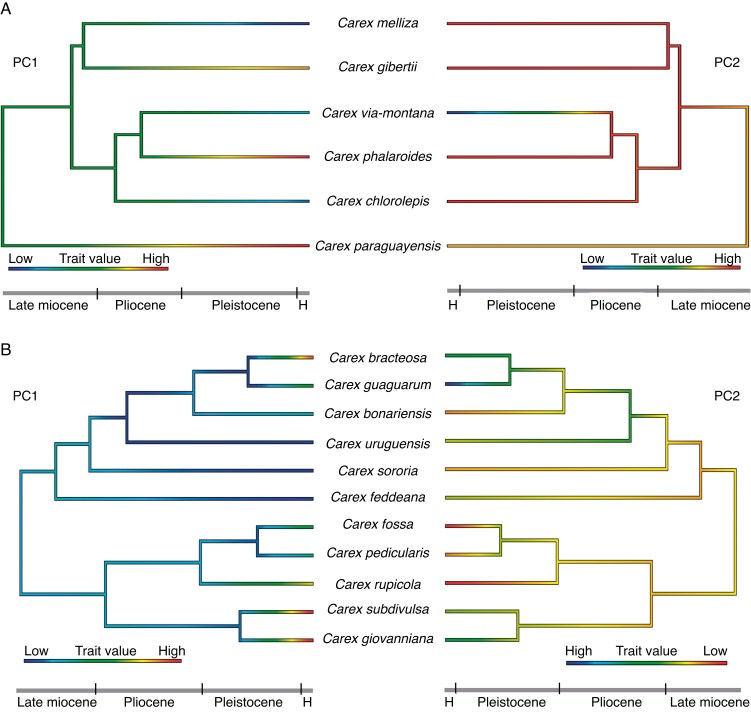
Ancestral niche reconstruction plot representing the bioclimatic variable PC values, PC1 and PC2. (A) *Carex phalaroides* group. (B) Sect. *Bracteosellae.* PC1 represents precipitation bioclimatic variables, while PC2 represents temperature bioclimatic variables. Within *C. phalaroides* group PC2, annual mean temperature has more importance while for sect. *Bracteosellae* the temperature seasonality variable has more importance. Under the reconstruction a simplified timeline is indicated.

Ancestral niche reconstruction was conducted using the Ornstein–Uhlenbeck model for both the *C. phalaroides* group and sect. *Bracteosellae*, as this model had a lower AICc value compared with the Brownian motion model (AICc = 11.46/3.76 and 57.17/57.19, respectively). Reconstructions were performed by pruning the trees to include only the in-group. The bioclimatic niche reconstruction is summarized in Fig. 3A, with the PC1 and PC2 values plotted onto the phylogeny. The evolution of individual variables is detailed in Supplementary Data Fig. S6. In the case of the *C. phalaroides* group, the ancestral niche reconstruction using PC1 values (precipitation variables) reveals niche shifts within lineages which are appreciated in the external parts of the tree, with no niche changes regarding precipitation in the deep nodes. Meanwhile, ancestral niche reconstruction using PC2 values (temperature variables) reveals a deep node niche shift, particularly evident in the divergence of *C. paraguayensis*. Additionally, *C. via-montana* exhibits a temperature shift unique to its branch, distinguishing it from the rest of the group. *Carex paraguayensis*, *C. gibertii* and *C. phalaroides* exhibit similar values on PC1, indicating higher annual precipitation with greater seasonality. Additionally, they display higher temperature values with lower seasonality. Notably, for precipitation, a clear niche shift is observed between eastern and western clusters. For the western cluster, reconstruction indicates similar bioclimatic conditions characterized by lower precipitation values and higher temperatures. However, *C. via-montana* stands out by displaying lower values for the temperature variables (Fig. 3A, Supplementary Data Fig. S6).

Individual bioclimatic variables for sect. *Bracteosellae* are available in Supplementary Data Fig. S7. Ancestral niche reconstruction for sect. *Bracteosellae* detected niche shifts towards the tips in both the PC1 and PC2 plots (Fig. 3B). For PC1 values, a shift towards higher values occurred synchronously in the sister species *C. giovanniana* and *C. subdivulsa* only after their divergence. Another shift was observed in the *C. bracteosa* lineage relative to its sister species. For PC2 values, the *C. guaguarum* lineage was the only detected shift across the phylogeny.

## DISCUSSION

Given the large scales considered in the present study, with two study groups with large distributions across South America, we take a macroecological approach and primarily consider geoclimatic events to explain the patterns we observe. This is a common methodological approach when addressing the biogeographical history of plant groups with wide distributions (see for example Luebert and Weigend, 2014; Benítez-Benítez *et al.*, 2021; Masa-Iranzo *et al.*, 2021; Bacci *et al.*, 2022; among many others). Although we have attempted to consider the competent macroecological framework at the present time, we cannot rule out the possibility that other smaller-scale factors may be playing relevant roles in shaping their current distributions (i.e. soil pH, salinity or nutrient content, biological interactions, topographic conditions, etc.).

### Biogeographical patterns of the *C. phalaroides* group

Within South America, *Carex* sect. *Junciformes* exhibits its highest diversity in Patagonia, where species tend to occupy a relatively conserved ecological niche, primarily associated with low-temperature environments (Benítez-Benítez *et al.*, 2021). This pattern of niche conservatism suggests that the expansion of sect. *Junciformes* beyond Patagonia was likely facilitated by the cooling of the low-latitude Andes during the Pliocene, which created new suitable habitats at higher elevations (Roberts *et al.*, 2017). This climatic shift may have enabled a northward expansion along the Andes, acting as a corridor towards tropical latitudes for several species (e.g. *C. camptoglochin*, *C. vallis-pulchrae*, *C. sanctae-marthae*; Benítez-Benítez *et al.*, 2021; Morales-Alonso *et al.*, 2024*a*).

Biome boundaries are known to act as significant constraints on lineage diversification and dispersal (Higgins *et al.*, 2016). In *Carex*, shifts in diversification rates have been linked to global cooling events at both deep and shallow evolutionary timescales (Spalink *et al.*, 2016; Martín-Bravo *et al.*, 2019), which aligns with the genus’s predominantly cold-temperate distribution worldwide. These findings underscore the role of geoclimatic dynamics in shaping diversification patterns, particularly in regions like the Southern Cone, where environmental heterogeneity and historical climate fluctuations have played a central role in driving evolutionary processes.

In contrast to the rest of the section, the *C. phalaroides* group exhibited a shift in its climatic preferences towards warmer conditions during the Late Miocene–Early Pliocene (Fig. 3A, Supplementary Data Fig. S6; see also Fig. 2 in Benítez-Benítez *et al.*, 2021). Our BSM and RevBayes results support a broad ancestral range for the crown node of the group, followed by a gradual geographical fragmentation (Fig. 2A, Supplementary Data Fig. S3A). Together, these findings are consistent with a classic vicariance scenario, similar to those documented in other plant lineages from the region (Luebert *et al.*, 2020).

During the initial stages in the Middle Miocene, the formation of the Paranean Sea may have prompted the expansion of the *C. phalaroides* group’s crown node beyond Patagonia, which at the time supported savannah-like vegetation (Sede *et al.*, 2012). The probable increase in humidity associated with this marine incursion, contrasting with the surrounding dryer regions, may have created favourable conditions for a broad, presumably continuous east–west distribution extending into tropical and subtropical latitudes (Fig. 2A). The end of the Mid-Miocene Climate Optimum marked a gradual decline in temperatures and a shift towards increasing aridity. As marine transgressions such as the Paranean Sea receded during the Late Miocene, and with the full establishment of both SADDs, the east–west connectivity of the populations was disrupted. This likely led to the fragmentation and contraction of populations into humid refugial zones, particularly along the eastern Andes (Yungas) and the Atlantic coast.

The Andean uplift process together with other tectonic and climatic dynamics (see Introduction) continued having their effects during the Late Miocene and Pliocene, leading to the wSADD and Patagonian Steppe establishment. The last one seems like the probable cause of the group’s absence in such southern latitudes. On the west and south-west side of the *C. phalaroides* group distribution, lineages underwent geographical isolation due to ecological (wSADD) and geographical (Andes) barriers, followed by posterior adaptation to the local conditions. This completed the latter stages of the proposed vicariant scenario. From south to north, different sets of isolated populations led to different species of the complex: the most recent common ancestor (MRCA) of *C. chlorolepis* was left confined to the Pacific slopes of Chile, creating a southern Andean–southern Brazilian disjunction (Luebert and Weigend, 2014). *Carex melliza* remained in the Yungas, while the ancestor of *C. via-montana* managed to disperse northwards through the Andean range and colonize the northern Andes.

### Biogeographical patterns of sect. *Bracteosellae*

Colonization events from North America to South America have been reported for several lineages with widespread Andean distributions (e.g. *Astragalus*, *Lupinus*; Hughes and Eastwood, 2006; Scherson *et al.*, 2008). Most of these events happened through long-distance dispersal events (LDD) during recent geological times after the bioclimatic Andean corridor had already been established (i.e. at ∼5 mya, when the northern Andes reached near-modern elevations), and prompted the colonizing lineages to disperse southwards through mountain-hopping (Tribble *et al.*, 2024 ). With a more extensive population-level sampling within the species, it would be possible to test the mountain-hopping hypothesis for this clade in South America; however, we only aimed to resolve evolutionary processes within a South American scale. However, Martín-Bravo *et al.* (2019) estimated that *Carex* sect. *Bracteosellae* originated from a North American ancestor during the Middle to Late Miocene (9.6–9.2 mya). By this time, elevations in the northern Andes were still insignificant and most orogenic activity was still accumulated in the Patagonian and central Andes (Gregory-Wodzicki, 2000;Hoorn *et al*., 2010; Boschman, 2021 ). According to our DIVA-like analysis results, *Carex* sect. *Bracteosellae* begins its diversification within a wide range in the eastern Southern Cone, comprising the Atlantic region, Pampa and central Andes areas. Again, at that time the Paranean Sea would have prompted accurate conditions for the establishment of *Carex* in the area. A direct colonization from North America to the eastern Southern Cone through LDD may be the most probable event that led to sect. *Bracteosellae* diversification. Big leap dispersal events from the Northern Hemisphere to the Southern Hemisphere have been reported for plant lineages exhibiting an American amphitropical disjunction (Raven, 1963; Simpson *et al.*, 2017) and have been described for several lineages of *Carex* (Toivonen, 1979; Villaverde *et al.*, 2015, 2017). Furthermore, North America has been interpreted as the primary source of diversity for South American lineages in *Carex* (Martín-Bravo *et al.*, 2019; García-Moro *et al.*, 2022).

Once again, our BSM and RevBayes results support a vicariance-driven scenario for sect. *Bracteosellae*, characterized by a broad ancestral area followed by a gradual geographical fragmentation (Fig. 2B, Supplementary Data Fig. S3B). However, the evolutionary path of this section contrasts with that of the *C. phalaroides* group by three facts: (1) the divergence events occurred later in geological time; (2) lineages that initially diverged in allopatry appear to have come into secondary contact during the Pleistocene and Holocene; and (3) some lineages show evidence of having dispersed across established geoclimatic barriers, suggesting a more dynamic biogeographical history.

In sect. *Bracteosellae*, an important split within Clade A took place during the Pliocene at ∼4.75 mya, separating a central Andean subclade and a Pampean subclade. During this period, Patagonia already displayed a xeromorphic biome and the central and south-central Andes uplifted to near their current elevations (Garzione *et al.*, 2006; Lamb, 2015). As no niche shifts were detected during the main cladogenetic events (Fig. 3B, Supplementary Data Fig. S7), we infer a vicariant process where the population remained in similar environments in the central Andes and the Pampa while the permeability through the eSADD disappeared. These findings suggest the existence of functional biogeographical connections for our study group across an almost fully established eSADD during the Early Pliocene. However, by the Pliocene–Pleistocene boundary these connections appear to have been lost (Trujillo-Arias *et al.*, 2018), reinforcing the role of the eSADD as a significant barrier for these two lineages. The species that remained in the central Andes (*C. giovanniana* and *C. subdivulsa*) underwent a synchronous niche shift towards higher precipitation levels (Fig. 3B) following their divergence (1.65 mya), probably as result of local adaptation to Yungas Forest humid conditions.

Both biogeographical analyses placed the ancestor of Clade B of sect. *Bracteosellae* as eastern South American; however, they differed in the specific ancestral area. The DIVA-like analysis identified the Atlantic region as the most likely origin, while RevBayes retrieved the Pampa. This discrepancy may reflect the incomplete establishment of these biomes during the relevant time period, resulting in poorly defined boundaries between them. Our results show that colonization of western areas within Clade B began at the crown node of *C. bonariensis* (2.72 mya). The DIVA-like analysis inferred a broad ancestral range encompassing the Atlantic region, central Andes and northern Andes. In contrast, the RevBayes analysis (Fig. 2B) suggested a more restricted ancestral area, primarily comprising the Pampa and Atlantic region. A dispersal event by the MRCA of *C. bonariensis* occurred across the eSADD into the central Andes during the Middle Pleistocene, potentially facilitated by riverine corridors (Trujillo-Arias *et al.*, 2018). However, the RevBayes analysis recovered an uncertain ancestral area for the crown node of *C. bonariensis–C. guaguarum–C. bracteosa* (Fig. 2B), possibly reflecting a rapid range expansion of the group’s ancestor (Supplementary Data Fig. S3B), followed by vicariance driven by the intensification of the wSADD. The crown node of *C. guaguarum* and *C. bracteosa* was retrieved to occupy a combined ancestral area in the northern Andes and Chile, a result compatible with either vicariance (Fig. 2B) or LDD processes (Supplementary Data Fig. S3B). Dispersal patterns from Chile to the tropical Pacific, skipping the central Andean sector, have been studied in several groups of plants (see the ‘south-central Andean disjunction’ section in Luebert and Weigend, 2014). This pattern is also observed in other *Carex* lineages, particularly within subg. *Uncinia*, where it occurred at least four times, in the *C. delacosta*, *C. phleoides*, *C. erinacea* and *C. formula* lineages (García-Moro *et al.*, 2022).

### Evaluating synchrony in biogeographical events

Cladogenetic events inferred with RevBayes stochastic mapping in the two study groups (Fig. 2, Supplementary Data Fig. S3) can be divided into synchronous and asynchronous.

Only one species from each lineage, *C. via-montana* in the *C. phalaroides* group and *C. guaguarum* in sect. *Bracteosellae*, successfully colonized the northern Andes. For both species, a nearly synchronous colonization process is inferred, probably driven by the role of the Andean Cordillera in generating new habitats. As stated in Luebert and Weigend (2014) , during the Pleistocene new niches became available for colonization and diversification of a great variety of elements, both from within and outside the Neotropical region (Simpson, 1983; Sklenâr and Balslev, 2007). Some were due to the elevation of the Andes itself, others to the climatic consequences of it. *Carex via-montana* and *C. guaguarum* thus represent additional examples of the Andes acting as a biological corridor (Luebert and Weigend, 2014).

The vicariance-based interpretation proposed to explain the cladogenetic diversification of the two study groups in the Yungas also seems to display a degree of synchrony. On one hand, the MRCA of *C. melliza* likely occupied a broad range covering Yungas and the Atlantic Forest until the Late Pliocene. On the other hand, by the end of the Early Pliocene the crown node of *C. subdivulsa* and *C. giovanniana* remained in the central Andean region, following contraction from a previous wider distribution that also included the Pampa. The fragmentation of both continuous wider distributions and the loss of connectivity through the eSADD might have been driven by the increasing aridity. This climatic shift was likely the result of the Late Pliocene Glaciation (3.5 mya, Rabassa *et al.*, 2005) and the intensification of the Andean rain shadow, both of which contributed to the disruption of dispersal corridors and the isolation of lineages in humid refugia.

The most remarkable asynchronous cladogenetic event observed in both phylogenies is the colonization and subsequent speciation in Chile. For the ancestor of *C. chlorolepis*, a broad ancestral range spanning the Atlantic–Chilean area was inferred during the Early Pliocene. In contrast, the diversification of *C. bracteosa*’s MRCA is inferred to have occurred much later, during the Middle Pleistocene, from a wider ancestral range encompassing the Chilean northern Andes. Given the estimated divergence time of *C. chlorolepis*, it is likely that its ancestor reached its current distribution in optimal climatic conditions, with subsequent range fragmentation driven by Quaternary glaciation cycles and the establishment of the wSADD. As discussed in the previous section, the origin of *C. bracteosa* remains uncertain, as it can be a consequence of both vicariance from a wider range or via LDD (as is also the case for *C. guaguarum*). However, the divergence time estimated for *C. bracteosa* coincides with a period when the wSADD was already established. If vicariance is assumed, this would suggest that the wSADD was not an effective barrier to dispersal during the Middle Pleistocene, aligning with the well-studied great dispersal capacity of the genus *Carex* (Villaverde *et al.*, 2017).

Anagenetic events occurred almost synchronously in both the *C. phalaroides* group and sect. *Bracteosellae* species during the Pleistocene (Supplementary Data Fig. S3), primarily on the eastern side of the continent. However, the directionality of these movements differed between lineages. While the ancestor of *C. gibertii* dispersed from the Atlantic region to the Pampa, the ancestors of sect. *Bracteosellae* species (*C. feddeana*, *C. sororia* and *C. uruguensis*) migrated in the opposite direction, from the Pampa towards the Atlantic region. A few million years later, a similar pattern was repeated with *C. phalaroides* and *C. pedicularis.* Although it is common to associate Pleistocene diversification events with the Quaternary glaciations, following Rull (2020), we caution against attributing these patterns solely to glacial cycles. The most probable explanation is the absence of a well-defined geographical barrier between the Atlantic and Pampa biomes. Instead, a dynamic and shifting boundary, driven by climate instability, likely facilitated bidirectional dispersal, promoting species permeability across regions.

### Comparative niche evolution

The wide ecological space displayed by both the *C. phalaroides* group and sect. *Bracteosellae*, encompassing eastern and western groups of populations and climatic shifts among lineages, may indicate an ecological niche expansion followed by local adaptation processes. Lineage differentiation has been mediated by similar but not equal processes, with a great relevance of the vicariance process for both groups but also of LDD dynamics for sect. *Bracteosellae*.

For the *C. phalaroides* group, the resulting fragmented distribution in the southern part of the range led to the different diverging lineages occupying different conditions of the ecological spectrum of the group. The western lineages seem to prefer temperate–cold habitats with lower precipitation (Fig. 3, Table 1) associated with the Andean Cordillera. Interestingly, *C. via-montana* shows a bimodal distribution in the response curve of temperature seasonality (Supplementary Data Fig. S5A), meaning there are two groups of populations, one in a more stable climate with no great variations of temperature throughout the year, and another preferring a wider temperature range through the year. As the species only displays this distribution in one of the climatic variables, we cannot confirm the existence of two *C. via-montana* ecotypes yet. Meanwhile, eastern *C. phalaroides* species are better suited to warm, humid habitats, especially *C. paraguayensis*. *Carex phalaroides*, although still preferring areas with high precipitation and temperatures, has a wider niche (Fig. 3, Table 1). Within the eastern lineages, *C. gibertii* had managed to occupy drier areas, being able to colonize the driest habitats in Pampa (Fig. 3, Table 1).

Section *Bracteosellae* lineages do not display these clear eastern–western lineages in bioclimatic variable division, as does the *C. phalaroides* group. This is probably due to the broader distribution of the group through Atlantic and Pampa regions. However, similarly, western lineages seem better suited to temperate conditions and lower precipitation when compared with the eastern lineages. We found two exceptions to this pattern (Fig. 3, Table 2): *C. guaguarum*, which shows a temperature optimum similar to *C. fossa*, a Pampa species; and *C. bracteosa*, with a precipitation requirement much higher than the rest of the western species. Eastern–western niche overlap and equivalency within taxa of the same lineage had already been tested in Luebert *et al.* (2020), who found that temperature shifts between eastern and western niches may be associated with the adaptation of Andean species to cooler conditions.

Comparative analysis between groups revealed that the northern Andes species *C. via-montana* and *C. guaguarum* recovered similar values for the analysed bioclimatic variables (Fig. 3, Supplementary Data Figs S6 and S7). The only exception was annual mean temperature, with *C. guaguarum* occurring in warmer temperatures than *C. via-montana*, suggesting that this species is better adapted to consistently warm conditions throughout the year. In contrast, the central Chilean species *C. chlorolepis* and *C. bracteosa* showed a slightly different pattern (Fig. 3, Supplementary Data Figs S6 and S7). While both species shared similar annual mean temperatures, *C. chlorolepis* experienced higher temperature seasonality, meaning that this species prefers environments with greater temperature oscillations throughout the year. Annual precipitation and precipitation in the driest quarter were comparable between species, although *C. bracteosa* showed slightly higher annual precipitation values. Despite differences in ancestral areas, biogeographical routes and diversification processes, extant lineages occupying the same regions tend to exhibit similar ecological preferences. This pattern suggests a process of bioclimatic niche convergence.

## Conclusions

The diversification of these two South American sedge lineages was driven by major geoclimatic events affecting South America since the Middle Miocene. We detected both cladogenetic and anagenetic processes, acting both synchronously and asynchronously during their evolutionary history. Additionally, despite their similar biogeographical trajectories, both groups display a wide ecological range. Notably, they also show evidence of bioclimatic niche convergence, suggesting that similar environmental pressures in overlapping regions have led to the evolution of comparable ecological preferences.

The favouring conditions during the Mid-Miocene Climate Optimum in the Southern Cone, such as marine transgressions, may have prompted the diversification and rapid expansion of the *C. phalaroides* group. This group initially occupied a broad range, which was subsequently fragmented during the Pliocene, resulting in two ecologically well-defined groups: one in eastern and one in western South America. Eastern lineages *C. phalaroides* and *C. gibertii* independently expanded their distributions from the Atlantic region into the Pampa. Western lineages managed to diversify in central Chile (*C. chlorolepis*), Yungas (*C. melliza*) and the northern Andes (*C. via-montana*) as a result of vicariance due to geoclimatic processes such as the SADDs, the final stages of the Andean uplift or the Quaternary glaciation cycles. The *C. phalaroides* group exhibits a clear niche shift between eastern and western lineages, especially for the only Andean species of the group, *C. via-montana*.

Diversification of sect. *Bracteosellae* began with an LDD event from the Northern Hemisphere and, once in South America, it spread through central Andes, Pampa and the Atlantic regions. We detected a vicariance process within Clade A at both sides of the eSADD, followed by a niche shift between species. A rapid expansion across the wSADD was also identified for *C. bonariensis*. The divergence of central and northern Andean lineages occurred during the Pleistocene, with the ancestral area retrieved as a combination of both regions. This pattern is consistent with either vicariance or LDD processes. *Carex bracteosa* remained confined to central Chile, while *C. guaguarum* expanded its range towards the recently established northern Andes, following a potential dispersal route facilitated by emerging ecological corridors.

The synchronous range expansions between the Atlantic region and the Pampa observed in both groups during the Pleistocene remain without a definitive explanation, though they may be linked to climatic fluctuations associated with Quaternary glaciation cycles. Another synchronous pattern is the differentiation of central Andean lineages in both groups, likely driven by vicariance processes. The availability of new habitats within the Andes Cordillera prompted the synchronous diversification of the Andean lineages across both groups. In contrast, the differentiation of the central Chilean lineages represents the only asynchronous event in the *C. phalaroides* group. This divergence may be a consequence of vicariance or, in the case of sect. *Bracteosellae*, even LDD. Despite differences in timing and mechanisms, both groups display a wide ecological range, with extant lineages in the same regions showing notable ecological similarities. Although the biogeographical histories of the *C. phalaroides* group and sect. *Bracteosellae* are identical, the convergence of ecological traits among co-occurring lineages suggests a process of bioclimatic niche convergence.

## Supplementary Material

mcaf262_Supplementary_Data

## Data Availability

Data used to generate the results of the study are available in the supplementary data files online. Newly sequenced Hyb-Seq genome sequences already trimmed were deposited in GenBank under accession numbers SAMN46881665-SAMN46881691 and BioProject PRJNA1225250.

## SUPPLEMENTARY DATA

Supplementary data are available at *Annals of Botany* online and consist of the following. Table S1: material used in this work for Hyb-Seq analyses, indicating taxa, the country, the locality from which it was collected, and the identification code (sample or extraction code). Voucher or collector(s) name (s) and number are indicated as in the herbarium label. The GenBank ID is also specified for the biosample and bioproject. Table S2: occurrence matrix for biogeographical analysis performed on BioGeoBEARS (Matzke, 2018). Areas are (1) A, Atlantic region; (2) P, P& (3) C, central Andes; (4) W, western flanks of the southern Andes (Chile); (5) N, northern Andes; and (6) O, others. Table S3: dataset for bioclimatic niche analyses. Botanical country is indicated following Brummitt (2001). Values for bioclimatic variables were obtained with WorldClim in Rstudio. Table S4: HybPiper statistics obtained for all the samples analysed. Table S5: HybPiper sequence lengths for all the samples analysed. Table S6: AMAS summary for all the gene matrices obtained for *C. phalaroides* group analyses. Table S7: AMAS summary for all the gene matrices obtained for sect. *Bracteosellae* analyses. Table S8: principal component summary and values for the uncorrelated bioclimatic variables analyses. Results are given for the *C. phalaroides* group (*Psyllophorae*) and sect. *Bracteosellae* (*Vignea*). Figure S1: phylogenomic reconstruction with coalescence approach using ASTRAL III. Branch support is indicated using PP above branches and SH-aLRT/UFBoot below branches when PP and UFBoot are <1 and 100, respectively. Bold branches indicate a maximum PP and UFBoot value. Quartet distance from every gene tree to the concatenated tree is represented by node pie charts, in black the percentage of the reference topology recovered, first alternative in grey, and second alternative in white. (A) *Carex phalaroides* group; (B) sect. *Bracteosellae*. Figure S2: divergence time estimation analysis in the *C. phalaroides* group (A) and sect. *Bracteosellae* (B) showing 95 % highest posterior density intervals. Figure S3: marginal character stochastic mapping from the Bayesian dispersal–extinction–cladogenesis biogeographical analysis for (A) the *C. phalaroides* group and (B) sect. *Bracteosellae*. Figure S4: PCA scatter plot of selected uncorrelated bioclimatic variables and its influence on species cluster formation. (A) *C. phalaroides* group, and (B) sect. *Bracteosellae*. Bio 1, annual mean temperature; Bio 4, temperature seasonality; Bio 12, annual mean precipitation; Bio 15, precipitation of the driest quarter; Bio 18, precipitation of the warmest quarter. Western and eastern species separation is indicated in the *C. phalaroides* group plot. Figure S5: comparison of density plots expressing the frequency of distribution of each uncorrelated selected variable in species belonging to (A) the *C. phalaroides* group and (B) sect. *Bracteosellae*. Figure S6: ancestral niche reconstruction of the *C. phalaroides* group based on the species tree representing the five uncorrelated bioclimatic variables selected. Figure S7: ancestral niche reconstruction of sect. *Bracteosellae* based on the species tree representing the five uncorrelated bioclimatic variables selected.
