# Supplementary material for: A tale of two lineages: comparative biogeography across the Southern Cone of two South American Carex (Cyperaceae) groups
Source: Ann Bot. 2025 Oct 22;138(2):368–86. doi: 10.1093/aob/mcaf262 (PMC13454685; doi:10.1093/aob/mcaf262)

Figure S1. Phylogenomic reconstruction with coalescence approach using ASTRAL III. Branch support is indicated using PP above branches and SH-aLRT/UFBoot below branches when PP and UFBoot < 1 and 100, respectively. Bold branches indicate a maximum PP and UFBoot value. Quartet distance from every gene tree to concatenated tree is represented by node piecharts, in black the percentage of the reference topology recovered, first alternative in grey, and second alternative in white. A: *C. phalaroides* group; and B: sect. *Bracteosellae*.

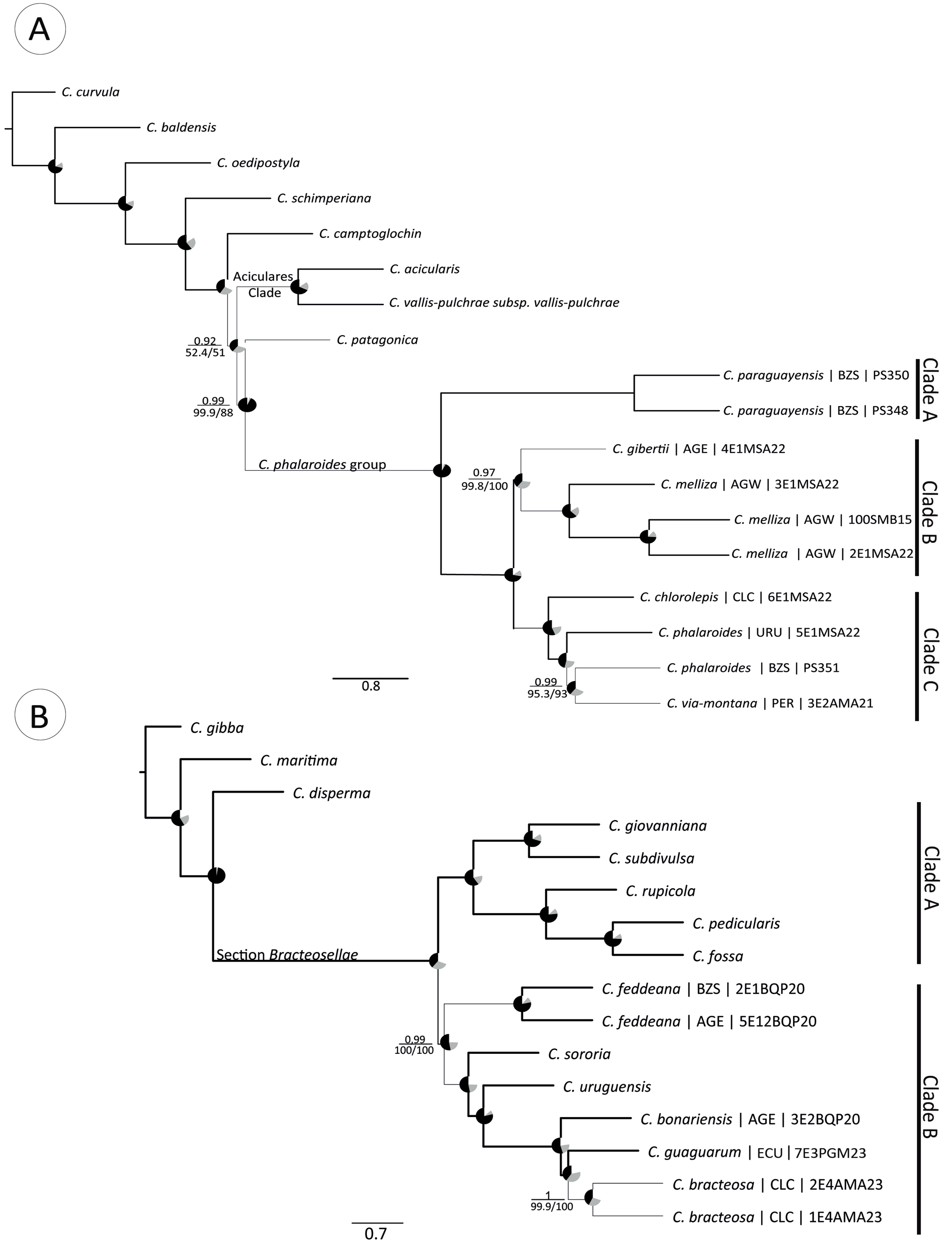

Figure S2. Divergence time estimation analysis in *C. phalaroides* group (A) and sect. *Bracteosellae* (B) showing 95% highest posterior density intervals.

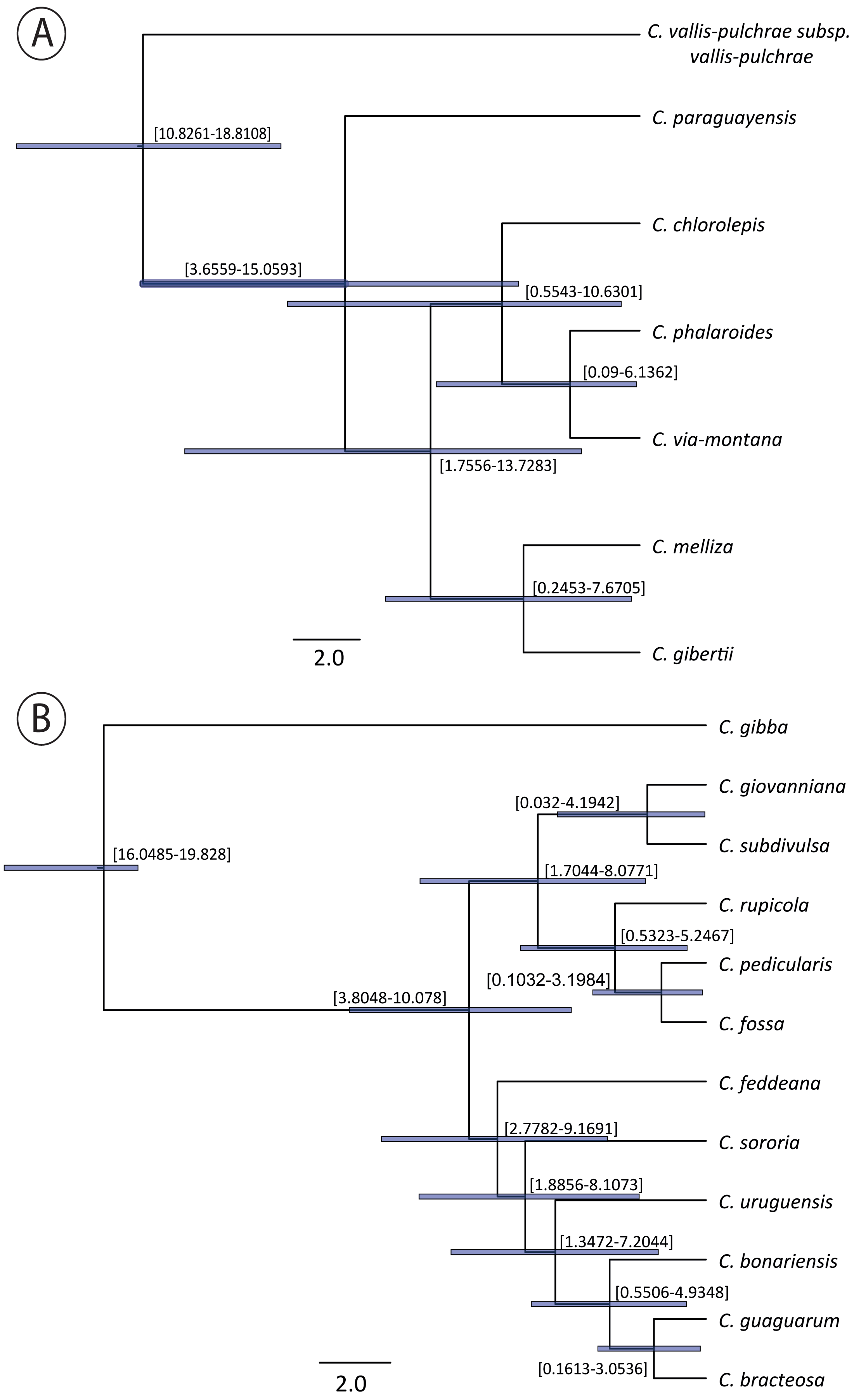

Figure S3. Marginal character stochastic mapping from the Bayesian Dispersal-Extinction-Cladogenesis biogeographic analysis for: A, the *C. phalaroides* group and B, sect. *Bracteosellae*.

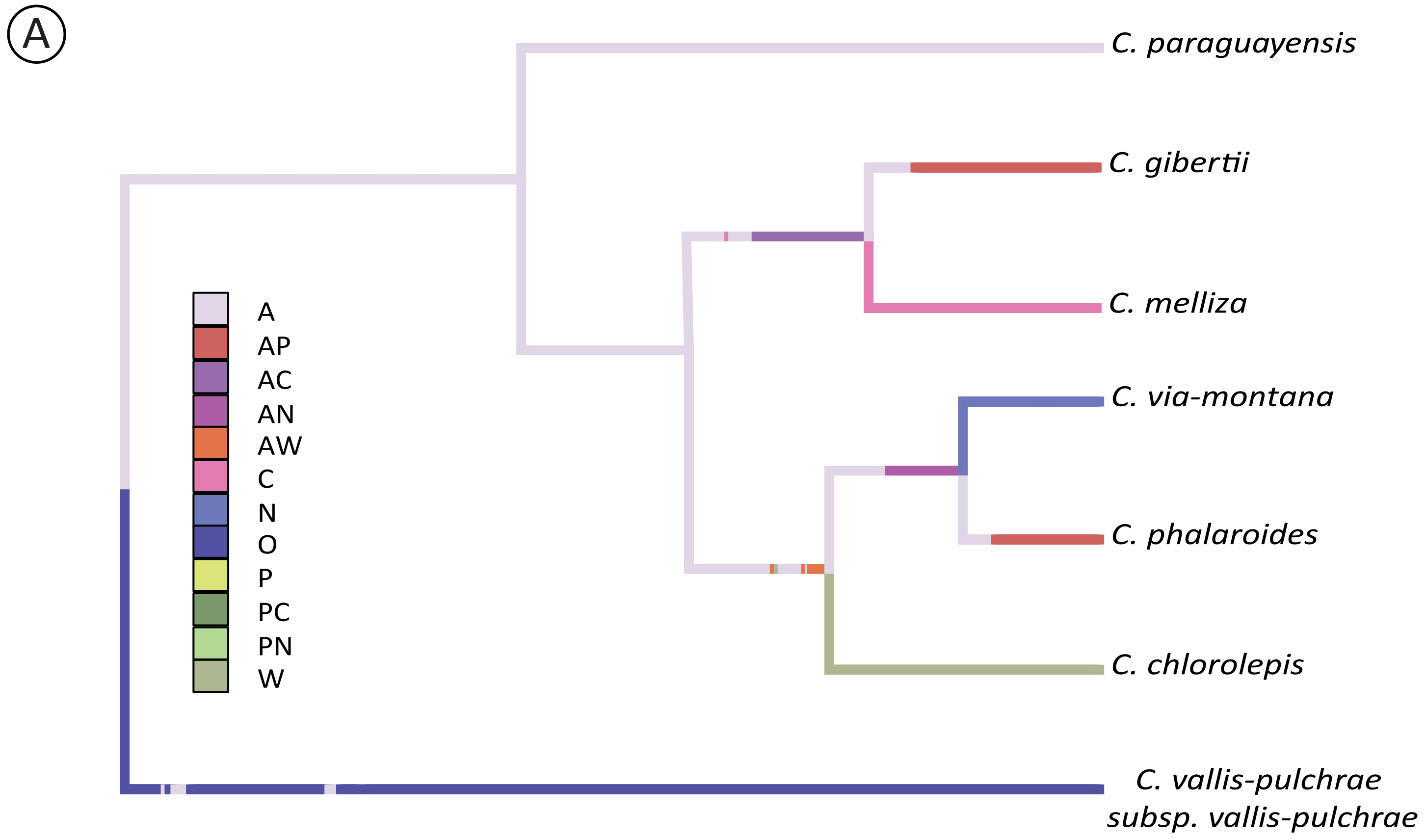

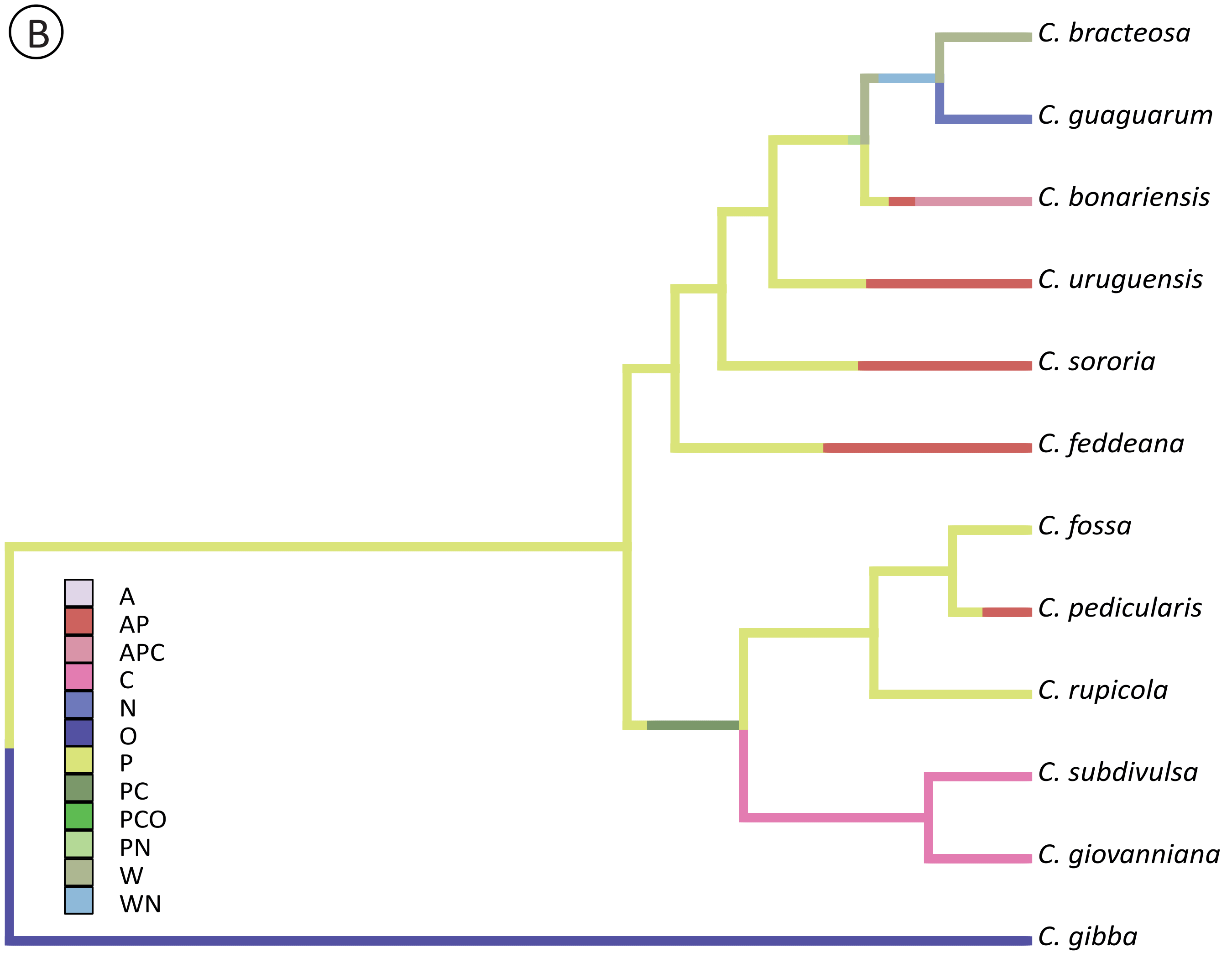

Figure S4. PCA scatter-plot of selected uncorrelated bioclimatic variables and its influence on species cluster formation. A, *C. phalaroides* group, and B, sect. *Bracteosellae*. BIO 1, annual mean temperature; BIO 4, temperature seasonality; BIO 12, annual mean precipitation; BIO 15, precipitation of the driest quarter; and BIO 18, precipitation warmest quarter. Western and eastern species separation are indicated in the *C. phalaroides* group plot.

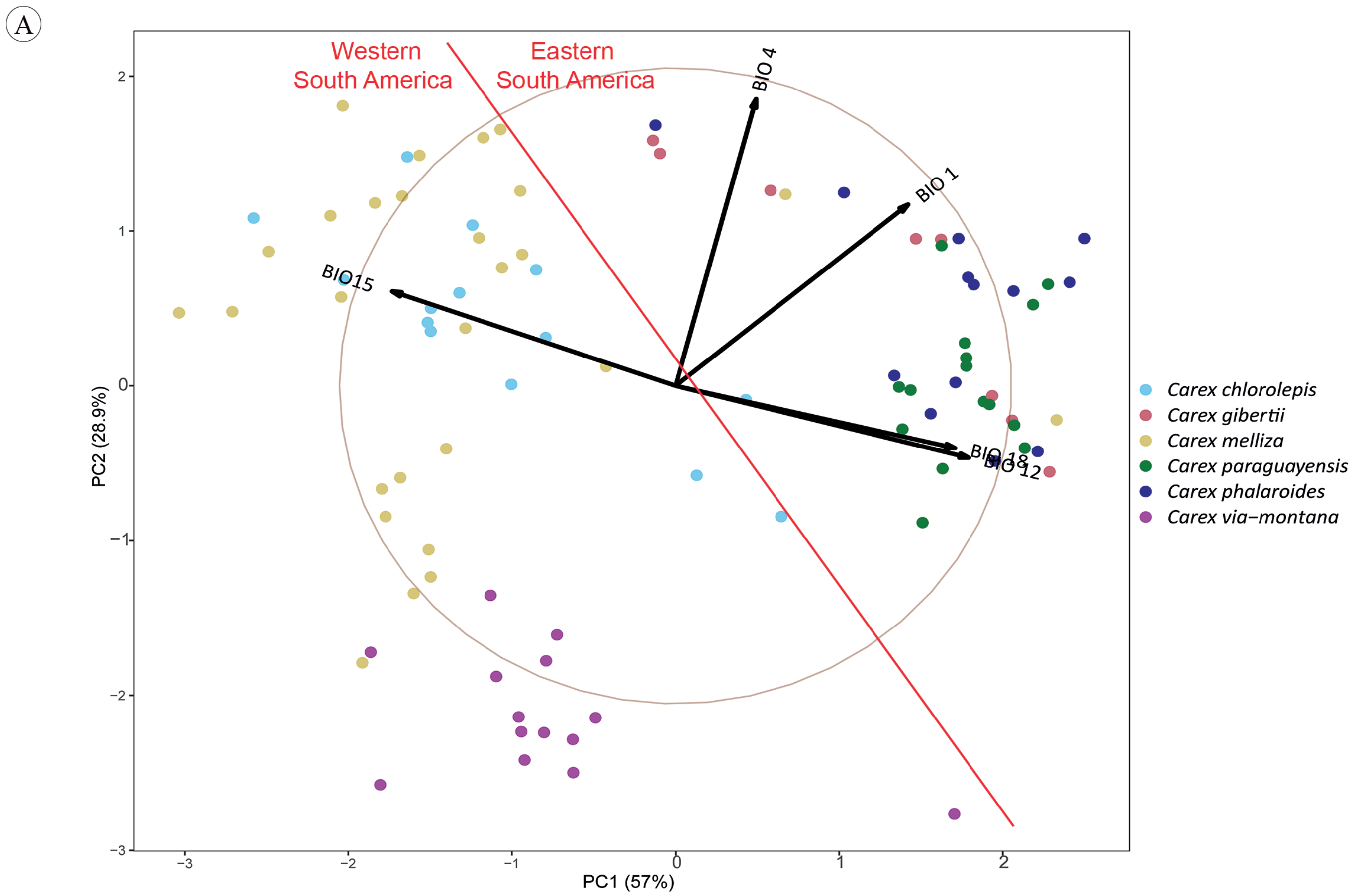

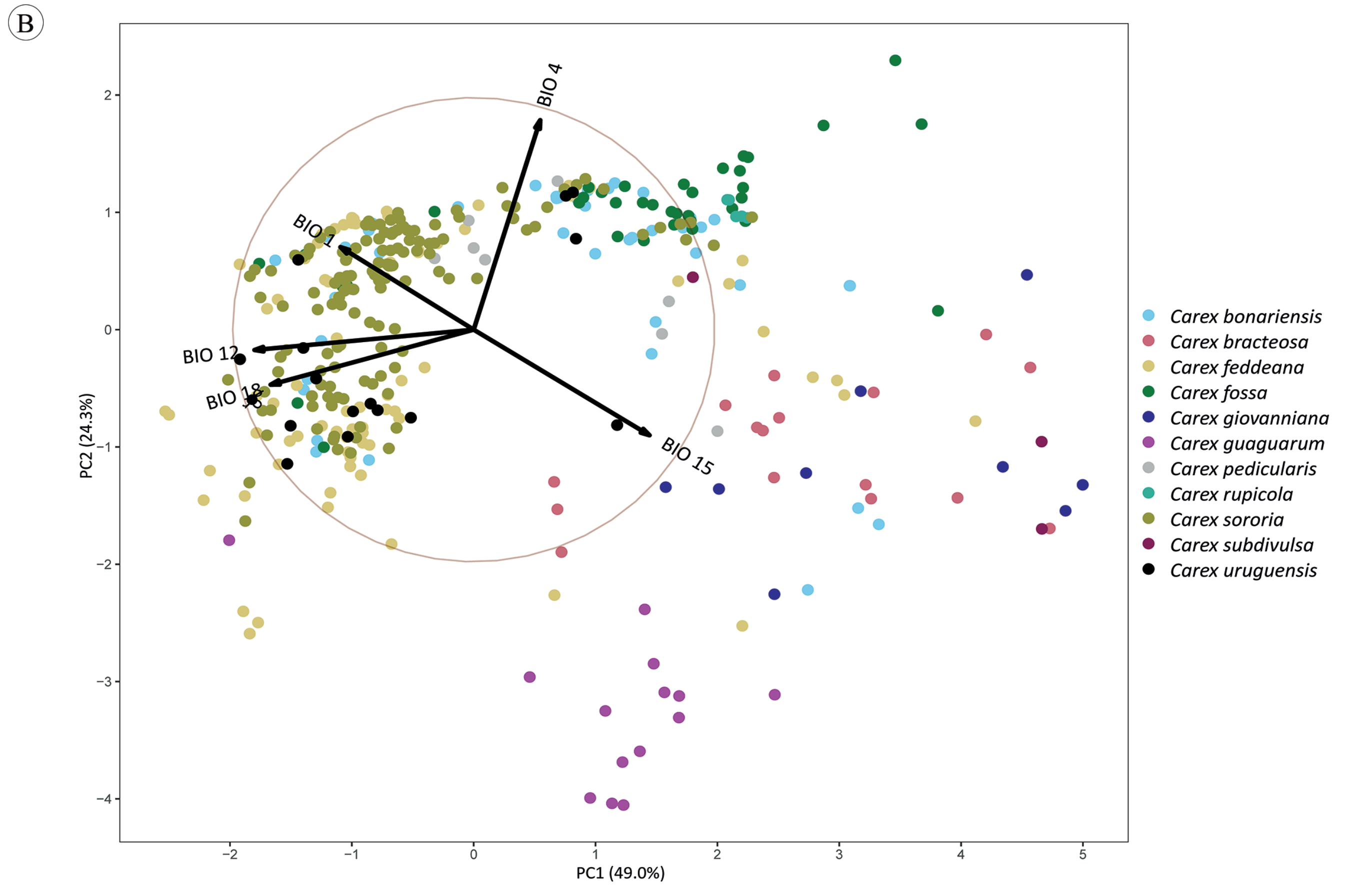

Figure S5. Comparison of density plots expressing the frequency of distribution of each uncorrelated selected variable in those species belonging to: (A) *C. phalaroides* group and (B) sect. *Bracteosellae*.

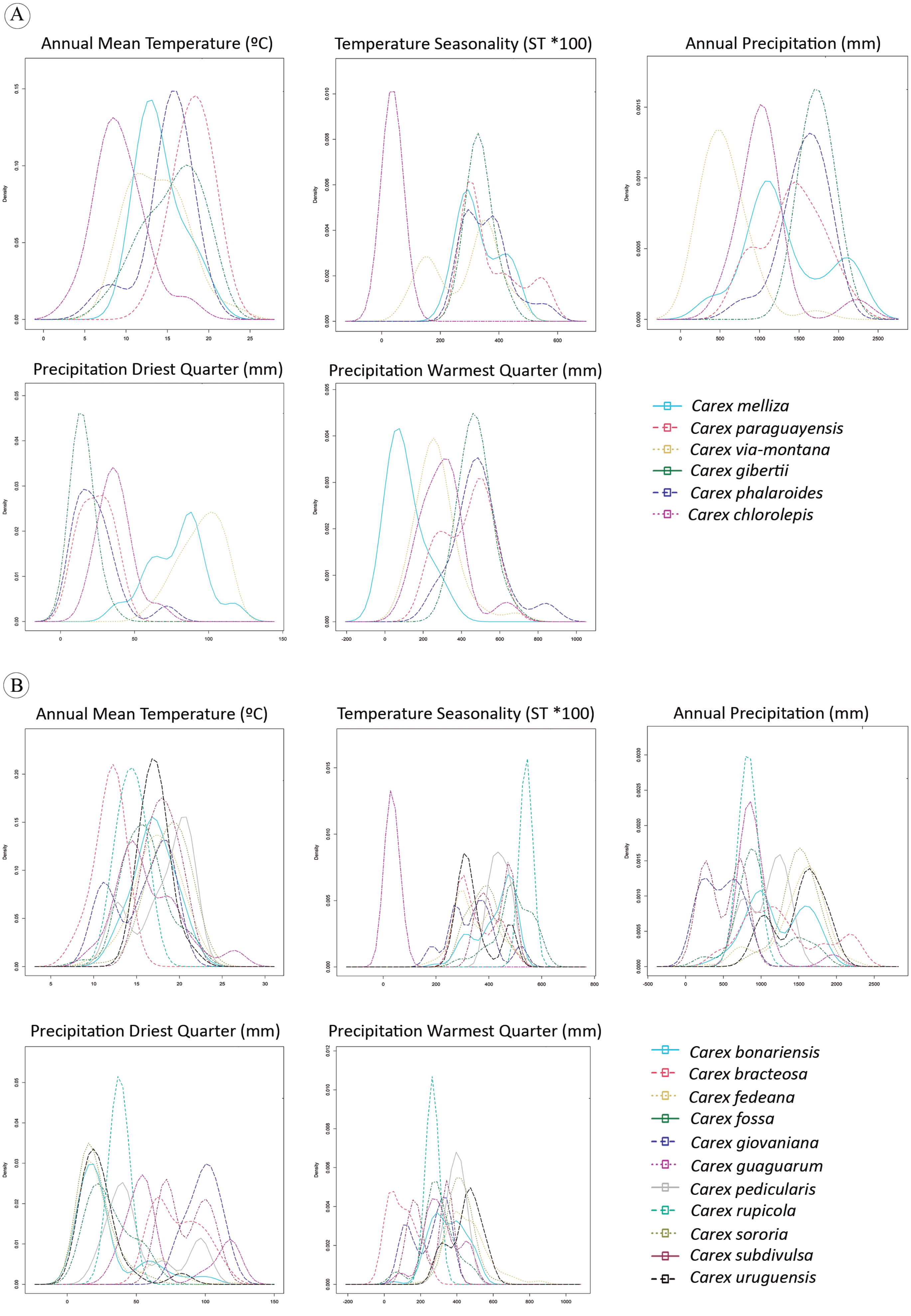

Figure S6. Ancestral niche reconstruction of the *C. phalaroides* group based on the species tree representing the five uncorrelated bioclimatic variables selected.

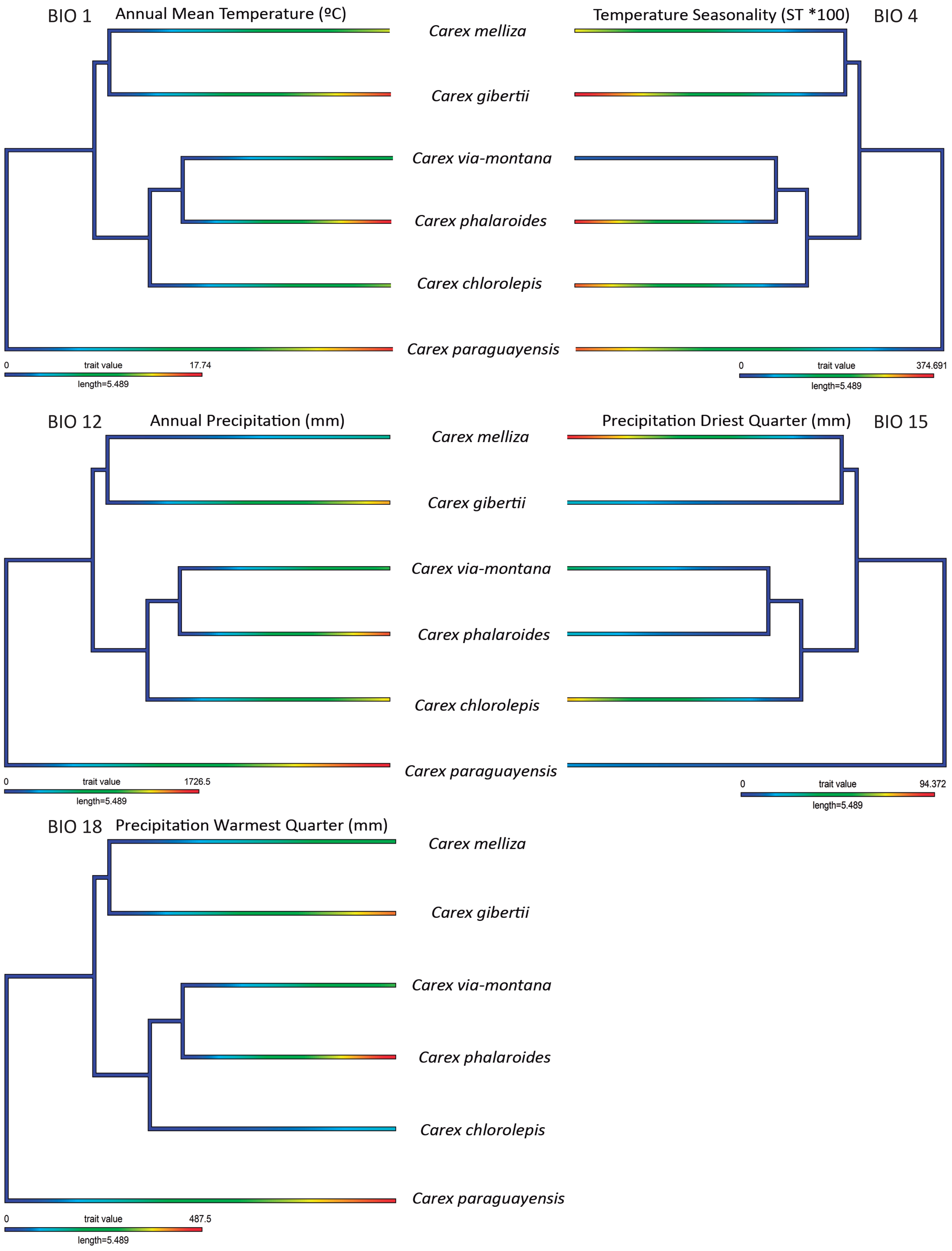

Figure S7. Ancestral niche reconstruction of the sect. *Bracteosellae* based on the species tree representing the five uncorrelated bioclimatic variables selected.

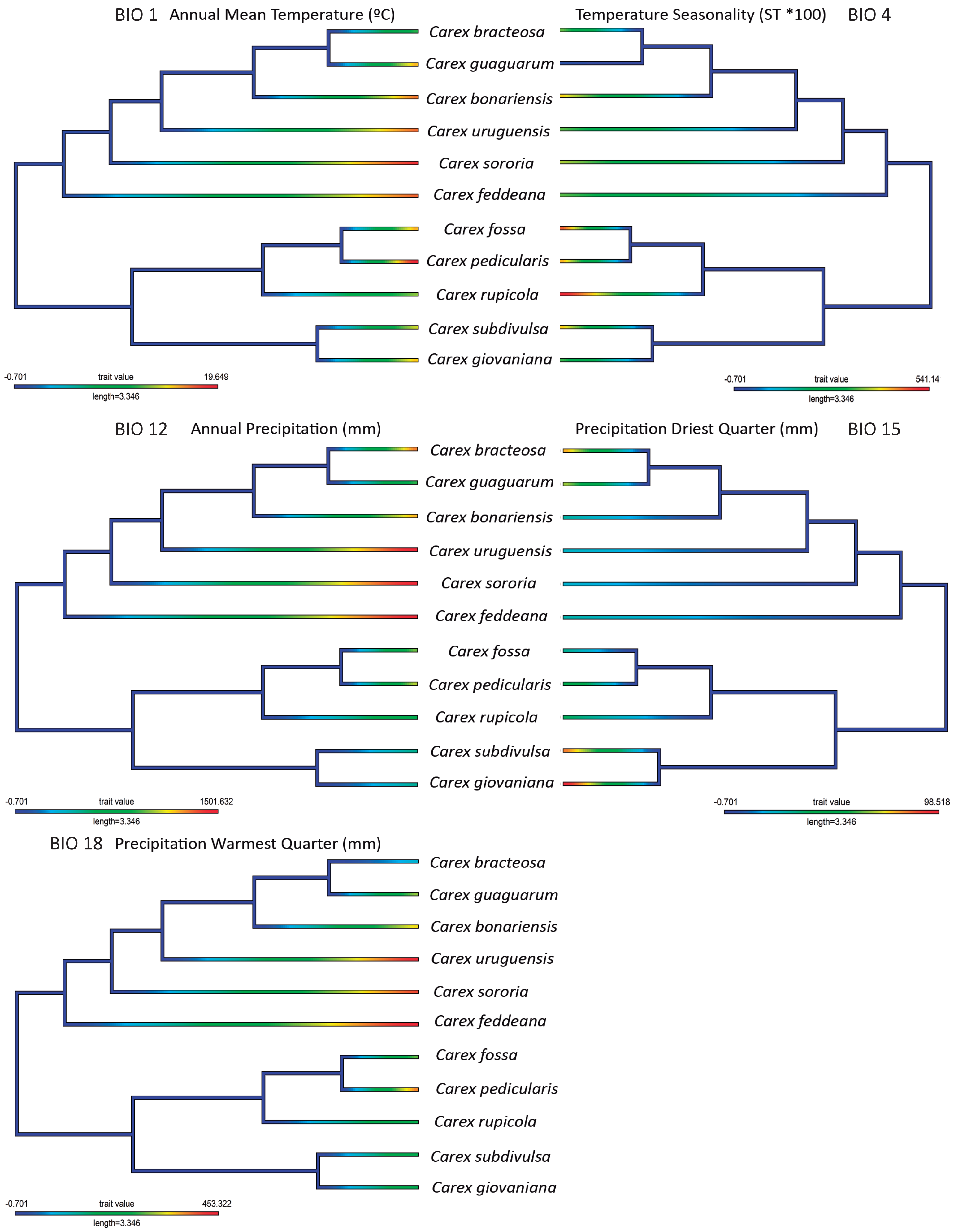

Supplement: Supplementary file 1 [file mcaf262_supplementary_data.zip › Supplementary_Figures.pdf]
